# Women’s neuroplasticity during gestation, childbirth and postpartum

**DOI:** 10.1038/s41593-023-01513-2

**Published:** 2024-01-05

**Authors:** María Paternina-Die, Magdalena Martínez-García, Daniel Martín de Blas, Inés Noguero, Camila Servin-Barthet, Clara Pretus, Anna Soler, Gonzalo López-Montoya, Manuel Desco, Susana Carmona

**Affiliations:** 1 Instituto de Investigación Sanitaria Gregorio Marañón, Madrid, Spain; 2 CIBER de Salud Mental, Instituto de Salud Carlos III, Madrid, Spain; 3 Departamento de Bioingeniería, Universidad Carlos III de Madrid, Madrid, Spain; 4 Unitat de Recerca en Neurociéncia Cognitiva, Departament de Psiquiatria i Medicina Legal, Universidad Autònoma de Barcelona, Barcelona, Spain; 5 Fundació IMIM, Barcelona, Spain; 6 Faculty of Health Science, Universidad Internacional de La Rioja (UNIR), La Rioja, Spain; 7 Centro Nacional de Investigaciones Cardiovasculares (CNIC), Madrid, Spain

**Keywords:** Cognitive neuroscience, Social neuroscience, Brain

## Abstract

Pregnancy is a unique neuroplastic period in adult life. This longitudinal study tracked brain cortical changes during the peripartum period and explored how the type of childbirth affects these changes. We collected neuroanatomic, obstetric and neuropsychological data from 110 first-time mothers during late pregnancy and early postpartum, as well as from 34 nulliparous women evaluated at similar time points. During late pregnancy, mothers showed lower cortical volume than controls across all functional networks. These cortical differences attenuated in the early postpartum session. Default mode and frontoparietal networks showed below-expected volume increases during peripartum, suggesting that their reductions may persist longer. Results also pointed to different cortical trajectories in mothers who delivered by scheduled C-section. The main findings were replicated in an independent sample of 29 mothers and 24 nulliparous women. These data suggest a dynamic trajectory of cortical decreases during pregnancy that attenuates in the postpartum period, at a different rate depending on the brain network and childbirth type.

## Abstract (web summary)

This longitudinal study tracked the brains of 139 first-time mothers. Mothers showed lower cortical volume before childbirth that attenuated during the postpartum, with a distinct recovery rate as a function of the brain network and birth type.

## Main

The transition to motherhood is a life-changing event. Pregnancy is marked by profound adaptations that affect almost every system of the mother’s body^1^. Recently, the brain has been recognized as an additional organ that adjusts its anatomy and function during gestation^2–9^. Backing up many years of animal research, the scarce but consistent literature regarding humans positions motherhood as a unique neuroplastic period in adult life^10^.

In humans, non-invasive brain imaging techniques such as magnetic resonance imaging (MRI) are leading the way in improving our understanding of pregnancy-related brain structural changes^11^. Prospective longitudinal MRI studies scanning mothers before and after their first pregnancy find cortical volume reductions in regions of the default mode network that remain for years after parturition^12,13^. Conversely, MRI studies scanning mothers across the postpartum period report an opposed trajectory, that is, cortical volume increases across multiple brain networks, including the default mode network^14–17^. Although seemingly contradictory, these findings fit with a dynamic evolution of brain volume changes comprising initial cortical declines during pregnancy followed by cortical increases during the postpartum period that do not attain pre-pregnancy levels, at least in default mode regions. In fact, 20 years ago, a study on pre-eclampsia that included nine healthy pregnant women as controls came across a similar trajectory when contouring the outer border of the brain^18^. However, whether pregnancy induces reductions in the cortical mantle that reverse during postpartum has not been empirically demonstrated.

Furthermore, to date, there is no solid evidence that the cortical volume reductions in the default mode network attributed to pregnancy are present before childbirth. Given that previous studies compared the brains of mothers before conception and 2–3 months into postpartum, it is possible that the observed decreases were induced by childbirth or even immediate postpartum factors.

The current neuroimaging study aims to unravel the specific contribution of pregnancy, childbirth and postpartum on maternal neuroplasticity. We reason that pregnancy and postpartum entail neuroplastic processes that translate into opposite effects on the cerebral cortex: reductions during pregnancy and increases during postpartum. We also propose that childbirth, an event with unique hormonal, immunological and physiological characteristics, may be the turning point at which the cortical trajectories reverse.

**Fig. 1:**
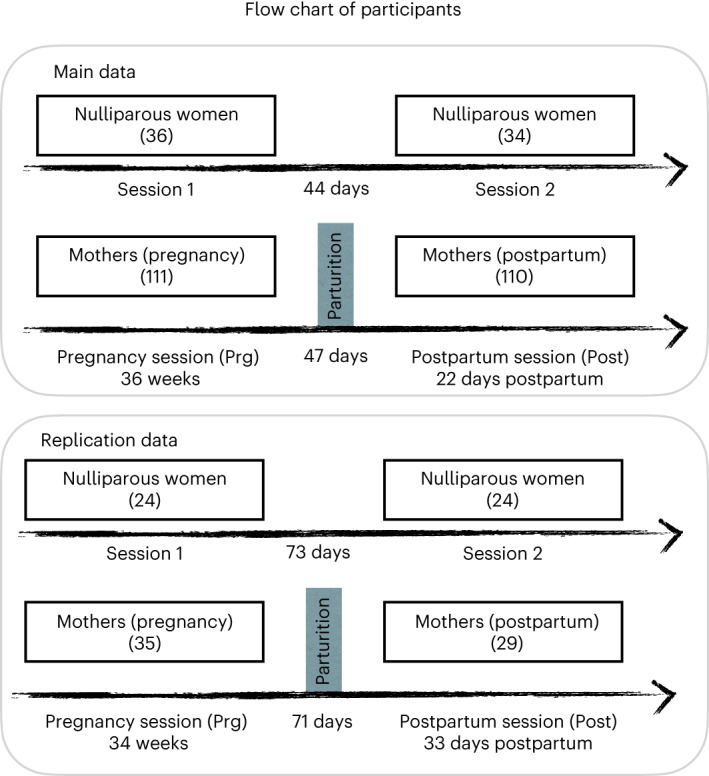
Diagram of participants. Number of mothers and nulliparous women participating in the first session during late pregnancy (Prg) and the second session during the early postpartum period (Post) and the drop-out between these sessions. In the main and replication datasets, three and six women were excluded because of loss of interest, respectively. Only participants who completed both Prg and Post sessions were included in the analyses.

By means of a case-control longitudinal design, we tracked the changes in cortical architecture from late pregnancy to early postpartum in the largest sample of first-time mothers explored so far, including a main sample of 110 mothers and a replication sample of 29 mothers. Both samples included a group of nulliparous women as controls (see the demographic information in Fig. 1 and Supplementary Table 1). Evaluations also included a wide range of obstetrics and neuropsychological variables to provide a comprehensive picture of the adaptations to motherhood. We first compared the brains of women in late pregnancy to those of nulliparous women to assess whether the previously reported cortical reductions are already present before parturition. Then, we determined whether the longitudinal changes from late gestation to early postpartum differ among groups. Next, we tested whether the longitudinal changes in mothers correlate with the percentage of postpartum time between the sessions to infer whether childbirth is the inflection point in the dynamic trajectory of cortical change. Finally, we evaluated whether childbirth modulates these brain trajectories by comparing the longitudinal changes in mothers who initiated labor (that is, experienced regular and intense uterine contractions and started cervical dilatation) with those who did not (that is, had a scheduled cesarean section (C-section)). This study helps to draw the dynamic trajectories of cortical change across motherhood and provides preliminary evidence that the type of childbirth impacts this trajectory.

In this paper, we use the term ‘women’ to refer to females whose sex matches their gender and ‘mothers’ to refer to females who were pregnant and gave birth to their children, in keeping with current practices in the field of parental neuroscience. This terminology will need to evolve to be more inclusive as the field expands to include gestational people whose sex and gender do not match.

## Results

### Cross-sectional and longitudinal cortical differences

**Fig. 2:**
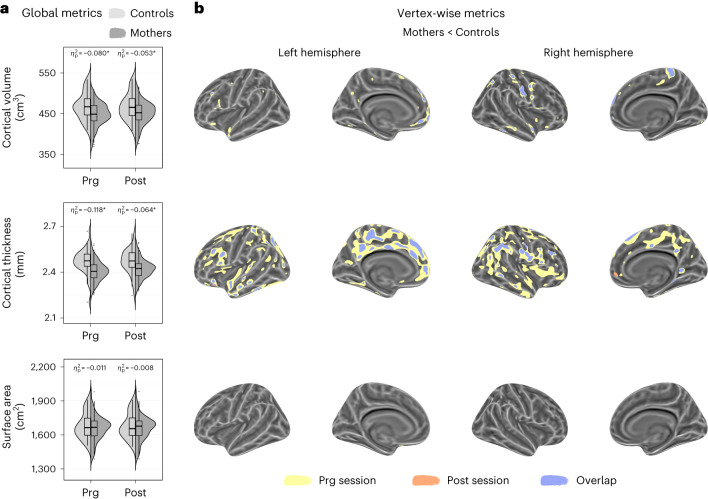
Group differences in cortical volume, thickness and surface area in mothers compared to nulliparous women at late pregnancy and early postpartum sessions. Group fixed effects at each session were studied through the adjusted linear mixed effect model: ${\rm{Cortical}}\,{\rm{Metric}} \sim 1+{\rm{Group}}+{\rm{Session}}+{\rm{Group}}\times {\rm{Session}}+\left(1| {\rm{Participant}}\right)$. **a**, Violin and embedded boxplots showing the distribution of the global cortical metrics at late pregnancy (Prg) and early postpartum (Post) sessions in mothers (*n* = 110) and controls (nulliparous women; *n* = 34). Effect sizes of the group differences in Prg and Post sessions were calculated as the signed partial eta squared (${\eta }_{p}^{2}$) associated with the corresponding one-tailed Wald *F*-tests. Asterisks indicate those differences surviving a *P* < 0.05 false discovery rate (FDR) correction. Exact *P* values are reported in Supplementary Table 2. The center line of the boxplot represents the median, the box encloses the lower and upper quartiles and the whiskers extend to the minimum and maximum values within a range of 1.5 times the interquartile range. Of note, the high inter-subject variability in surface area might have reduced the statistical power to detect group differences. **b**, Vertex-wise binary maps of the significant cortical differences between groups, specifically lower values in mothers compared to controls (FDR-corrected *P* < 0.05). Yellow, orange and blue indicate lower cortical values during Prg and Post sessions and the overlap between both sessions. Binary maps were projected to the inflated *fsaverage* template provided by the FreeSurfer software.

During late pregnancy (Prg), mothers displayed lower global cortical volume and thickness than controls (Fig. 2a and Supplementary Table 2). A vertex-wise analysis indicated that group differences mainly affected cortical thickness (Fig. 2b and Supplementary Fig. 1). These differences were widespread and included midline regions extending from the medial prefrontal gyrus into the anterior and posterior cingulate, and lateral areas including the precentral and postcentral sulci, the dorsolateral prefrontal cortex and the temporoparietal junction (Supplementary Table 3). In the postpartum session (Post), mothers also showed lower cortical volume and thickness than controls (Fig. 2a and Supplementary Table 2). Such differences overlapped with brain areas affected during pregnancy but were less extended (Fig. 2b, Supplementary Fig. 1 and Supplementary Table 4).

**Fig. 3:**
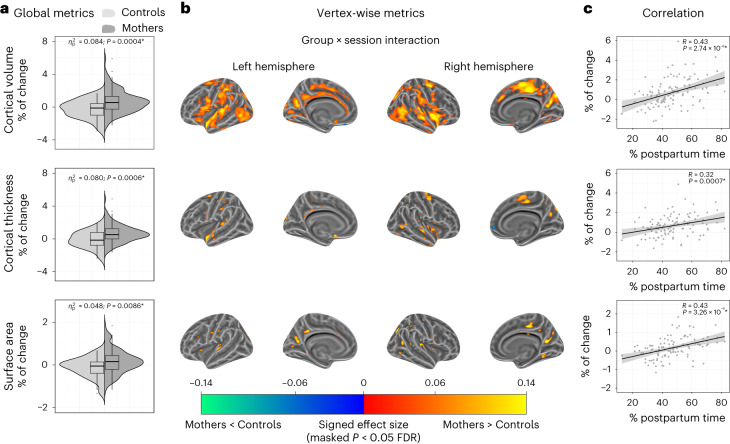
Longitudinal changes in cortical volume, thickness and surface area from late pregnancy to early postpartum in mothers compared to nulliparous women. Longitudinal changes were derived from the group × session interaction fixed effect term of the adjusted linear mixed effect model: ${\rm{Cortical}}\,{\rm{Metric}} \sim 1+{\rm{Group}}+{\rm{Session}}+{\rm{Group}}\times {\rm{Session}}+\left(1| {\rm{Participant}}\right)$. **a**, Violin and embedded boxplots showing the distribution of the percentage of change of the global metrics in mothers (*n* = 110) and controls (nulliparous women; *n* = 34). Effect sizes were calculated as the signed partial eta squared (${\eta }_{p}^{2}$) associated with the corresponding one-tailed Wald *F*-tests. Asterisks indicate those changes surviving a *P* < 0.05 false discovery rate (FDR) correction. The center line of the boxplot represents the median, the box encloses the lower and upper quartiles and the whiskers extend to the minimum and maximum values within a range of 1.5 times the interquartile range. **b**, Vertex-wise signed effect size maps (${\eta }_{p}^{2}$) of the group × session interaction (FDR-corrected *P* < 0.05), indicating larger decreases (blue) and larger increases (red) in mothers than in controls. Signed effect size maps were projected to the inflated *fsaverage* template provided by the FreeSurfer software. **c**, Correlations between the global percentages of change in cortical metrics and the percentage of postpartum time between sessions. The black line and the shaded area represent the least squares regression line and the 95% confidence intervals, respectively. Asterisks indicate two-tailed Pearson’s correlation coefficients (*R*) surviving an FDR-corrected *P* < 0.05. Uncorrected *P* values (*P*) below the threshold of 0.0001 are reported in exponential notation.

The group × session interaction term revealed significant Prg-to-Post longitudinal increases in global cortical volume, thickness and surface area in mothers compared to controls (Fig. 3a and Supplementary Table 2). At the vertex-wise level, Prg-to-Post cortical increases mainly affected cortical volume (Fig. 3b). These increases were widespread but primarily affected midline regions, including the posterior cingulate, the paracentral gyrus and the precuneus, and lateral areas, including precentral and supramarginal gyri and the superior temporal gyrus (Supplementary Table 5). We also identified Prg-to-Post cortical thickness and surface area increases in mothers, albeit to a lesser extent (Fig. 3b).

In mothers, the more the percentage of postpartum time between sessions, the greater the percentage of change in global cortical volume, thickness and surface area (Fig. 3c). Supplementary analyses revealed comparable results when accounting for the effect of potential confounding variables such as participants’ age, total intracranial volume and mean Euler number, as well as group differences in sleep quality (Pittsburgh Sleep Quality Index, PSQI) and perceived stress (Perceived Stress Scale, PSS) (Supplementary Figs. 2 and 3 and Supplementary Tables 6 and 7).

**Fig. 4:**
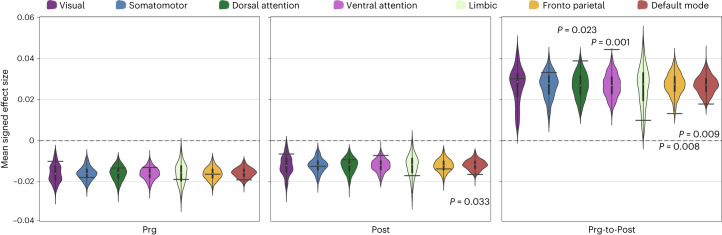
Spin testing for the mean signed effect sizes of the vertex-wise group differences in cortical volume. Group differences between mothers (*n* = 110) and nulliparous women (controls; *n* = 34) in cortical volume within the seven large-scale functional brain networks^19^. Black horizontal bars represent the observed values and the violin plots reflect the null distributions obtained using 1,000 spin-permutations of the maps. The exact one-tailed *P* values are reported when *P* < 0.05. No multiple comparison corrections were applied. The white dot on the center of the boxplot represents the median, the box encloses the lower and upper quartiles and the whiskers extend to the minimum and maximum values within a range of 1.5 times the interquartile range. Prg-to-Post, from pregnancy to postpartum sessions.

We assessed the spatial correspondence between the signed effect size maps of the vertex-wise analyses and the Yeo’s seven large-scale functional brain networks^19^ (Fig. 4). During late pregnancy, mothers showed lower cortical volume across all functional networks. However, at early postpartum, lower cortical volume in mothers was significantly more prominent in the default mode network. The group × session interaction term revealed significant above-chance cortical volume increases in attentional networks and significant below-chance cortical volume increases in frontoparietal and default mode networks. See Supplementary Fig. 4 for the spatial correspondence of cortical thickness and surface area maps.

**Fig. 5:**
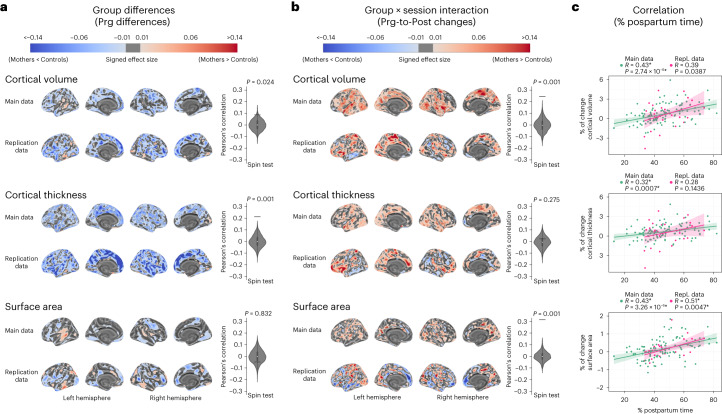
Comparison between the main and replication datasets. Main dataset comprised 110 mothers and 34 nulliparous women (controls); replication dataset comprised 29 mothers and 24 controls. **a**, Vertex-wise signed effect size maps of the cortical group differences indicating lower (blue) and higher (red) values in mothers compared to controls at late pregnancy (Prg). **b**, Vertex-wise signed effect size maps of the cortical longitudinal changes indicating larger decreases (blue) and larger increases (red) in mothers compared to controls. For **a** and **b**, signed effect size (${\eta }_{p}^{2}$) maps (collapsed to ±0.14) of the one-tailed Wald *F*-tests were projected to the inflated *fsaverage* template provided by the FreeSurfer software. Black outlines on brain surfaces enclose results surviving a false discovery rate (FDR)-corrected *P* < 0.05. Plots to the right of the maps test the spatial correspondence between the main and replication maps by means of one-tailed spin tests using Pearson’s correlation. Black horizontal bars represent the observed values and the violin plots reflect the null distributions obtained using 1,000 spin-permutations of the maps. The white dot on the center of the boxplot represents the median, the box encloses the lower and upper quartiles and the whiskers extend to the minimum and maximum values within a range of 1.5 times the interquartile range. **c**, Correlations between the global percentages of change in cortical metrics and the percentage of postpartum time between sessions in the main (green) and replication (pink) datasets. Colored lines and the shaded areas represent the least squares regression lines and the 95% confidence intervals. Asterisks indicate two-tailed Pearson’s correlation coefficients (*R*) surviving an FDR-corrected *P* < 0.05. Uncorrected *P* values (*P*) below the threshold of 0.0001 are reported in exponential notation. Prg-to-Post, from pregnancy to postpartum sessions; Repl., replication.

The main neuroanatomic findings were replicated in the independent dataset. Specifically, at late pregnancy, the main and replication datasets displayed above-chance spatial correspondence in cortical volume and thickness effect size maps (Fig. 5a). Furthermore, in the replication dataset, mothers also showed significantly lower cortical thickness in medial wall regions, despite the reduced number of subjects and, thus, lower statistical power. In the group × session interaction, above-chance spatial correspondence was observed for cortical volume and surface area (Fig. 5b). Finally, as in the main dataset, mothers in the replication sample displayed significant positive associations between the percentage of change in cortical volume and surface area and the percentage of postpartum time between sessions (Fig. 5c). A similar, albeit non-significant, association was observed between the percentage of change in cortical thickness and the percentage of postpartum time.

### Neuropsychological variables

In the group of mothers, perceived stress (PSS), sleep problems (PSQI), depression symptoms (Edinburgh Depression Scale) and maternal attachment (Maternal Attachment Scale) scores were higher during the postpartum session than the pregnancy session (Supplementary Table 8). Perceived stress and sleep quality significantly worsened from Prg to Post in mothers compared to controls (Supplementary Table 9).

**Fig. 6:**
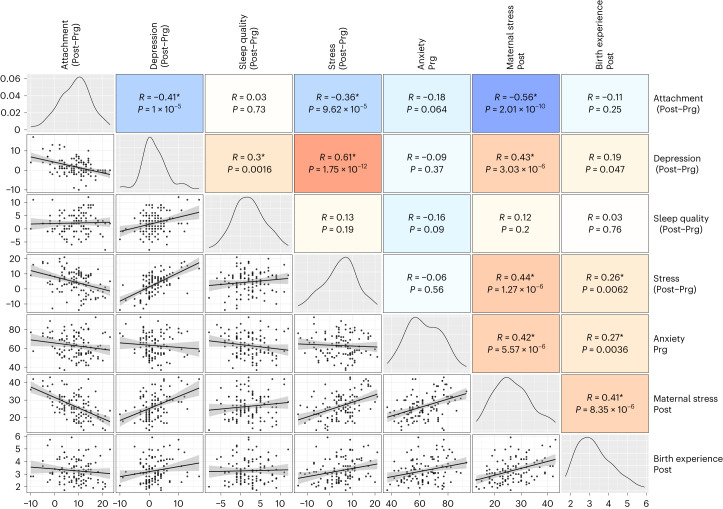
Correlation matrix of the neuropsychological variables in the mothers’ group (*n* = 110). The diagonal shows the distribution of the variables; the lower diagonal shows the scatterplots with the least square regression lines and the 95% confidence intervals, and the upper diagonal shows the Pearson coefficients (*R*) and associated uncorrected *P* values. Blue and red cells represent negative and positive correlations, respectively. Asterisks indicate Pearson coefficients surviving a two-tailed *P* < 0.05 FDR correction. *P* values below the threshold of 0.0001 are reported in exponential notation. From left to right, the neuropsychological variables correspond to the following questionnaires: Maternal Attachment Scale, Edinburgh Depression Scale, PSQI, PSS, Pregnancy Anxiety Scale, Maternal Stress Scale and Birth Experience Questionnaire.

Figure 6 shows the correlation matrix among the neuropsychological scores of the mothers. We found that higher scores of anxiety during pregnancy were associated with a worse birth experience and increased maternal stress about parenting during the postpartum session. A worse childbirth experience was associated with increased Prg-to-Post perceived stress as well as higher postpartum maternal stress. In turn, increased Prg-to-Post perceived stress and postpartum maternal stress were associated with Prg-to-Post increases in depression scores and decreases in maternal attachment. Lastly, increases in Prg-to-Post depression scores were linked to increases in sleep problems and decreases in maternal attachment. For a complete view of the neuropsychological correlations at the cross-sectional level, see Supplementary Fig. 5.

In the group of mothers, we assessed whether the neuropsychological measures were associated with the percentages of change in global cortical metrics. None of the explored correlations were significant (Supplementary Fig. 6).

### Cortical metrics as a function of childbirth

**Fig. 7:**
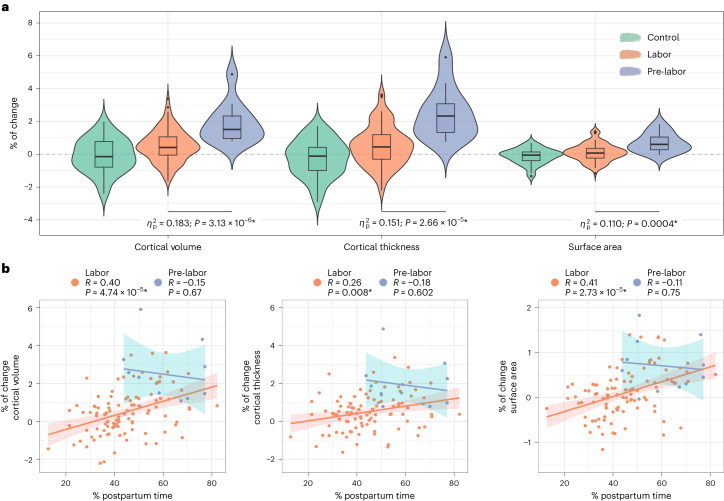
Longitudinal changes in cortical metrics in mothers who initiated labor and mothers who underwent a scheduled C-section. **a**, Violin and embedded boxplots showing the distribution of the global percentages of change in cortical volume, thickness and surface area. Longitudinal changes were derived from the group × session interaction fixed effect term of the adjusted linear mixed effect model: ${\rm{Cortical}}\,{\rm{Metric}} \sim 1+{\rm{Group}}+{\rm{Session}}+{\rm{Group}}\times {\rm{Session}}+\left(1| {\rm{Participant}}\right)$. Signed effect sizes were calculated as partial eta squared (${\eta }_{p}^{2}$) associated with the corresponding one-tailed Wald *F*-tests. Asterisks indicate results surviving a *P* < 0.05 false discovery rate (FDR) correction. Nulliparous women (control; *n* = 34) are displayed as a reference to mothers who initiated labor (*n* = 99) and mothers who underwent scheduled C-sections (pre-labor; *n* = 11). The center line of the boxplot represents the median, the box encloses the lower and upper quartiles and the whiskers extend to the minimum and maximum values within a range of 1.5 times the interquartile range. **b**, Correlations between the global percentages of change in cortical metrics and the percentage of postpartum time between sessions in mothers undergoing labor (orange) and pre-labor (purple). Colored lines and shaded areas represent the least squares regression lines and the 95% confidence intervals, respectively. Asterisks indicate two-tailed Pearson’s correlation coefficients (*R*) surviving a *P* < 0.05 FDR correction. For **a** and **b**, uncorrected *P* values (*P*) below the threshold of 0.0001 are reported in exponential notation.

We compared the cortical trajectories between mothers who initiated labor versus those who had a scheduled C-section and therefore did not initiate labor. Mothers who had a scheduled C-section displayed larger Prg-to-Post global increases in cortical volume, thickness and surface area compared to mothers who initiated labor (Fig. 7a and Supplementary Table 10). Prg-to-Post increases in these three cortical metrics persisted after correcting for gestational weeks at childbirth, the time between childbirth and the postpartum session, participant age, total intracranial volume and mean Euler number (Supplementary Table 11). None of these differences were detected at the vertex-wise level. Of note, neither at the pregnancy nor at the postpartum sessions did the mothers who underwent labor versus scheduled C-sections differ in global and vertex-wise cortical volume, thickness and surface area (Supplementary Table 10).

Post-hoc correlations between the percentage of postpartum time and the percentage of cortical change as a function of labor indicated that larger increases in the pre-labor group do not reflect a steeper slope of cortical increases as postpartum progresses (Fig. 7b).

Among those mothers who initiated labor, there were no significant differences in cortical volume, thickness or surface area between women who delivered vaginally and those who had an emergency C-section. Comparisons as a function of the delivery method are depicted in Supplementary Fig. 7 and Supplementary Table 12. For visualization purposes, we also show the data distribution of percentages of change in cortical metrics as a function of labor and delivery mode in the replication dataset (Supplementary Fig. 8).

## Discussion

In this neuroimaging study, we focused on the unexplored period of peripartum and provided evidence of a dynamic cortical brain trajectory accompanying the transition to motherhood. First-time mothers showed lower cortical volume and thickness before parturition, which attenuated during the postpartum period. The more the proportion of postpartum time, the larger the cortical increases from late pregnancy to early postpartum. Our data also suggest a different longitudinal cortical trajectory in mothers who delivered through a scheduled C-section. Throughout this discussion, we will refer primarily to cortical changes and will only differentiate between volume, thickness and surface area when necessary, such as when the results or prior literature apply to a specific metric.

The literature on the human maternal brain carries an ongoing debate as to whether the transition to motherhood entails decreases or increases in cortical volume. Studies capturing the pregnancy period report cortical reductions^12,13,20^, whereas studies that capture the postpartum window report the opposite pattern^14–17^. To date, the only two well-controlled longitudinal studies encompassing the whole pregnancy period compared the brains of first-time mothers between pre-conception and 2–3 months postpartum^12,13^. Such studies lacked within-pregnancy scans, which are necessary to disentangle the effects of pregnancy, parturition and early postpartum. In the present study, we assessed the peripartum period, including a late pregnancy scan, of the largest sample of first-time mothers studied to date. Our results provide solid evidence that cortical volume reductions in the maternal brain are present before childbirth, suggesting that they are, at least partially, triggered by pregnancy factors. The present study additionally found that pregnancy-induced cortical reductions are attenuated in the early postpartum session. Specifically, we found that the more the percentage of postpartum time between the sessions, the larger the cortical increases from late pregnancy to early postpartum. These findings reconcile data derived from pregnancy and postpartum studies, supporting the dynamic trajectory of cortical volume decreases during pregnancy that valley at around parturition and attenuate as postpartum progresses.

There is a broad consensus in the field of study involving the maternal brain that pregnancy-related cortical volume reductions are mostly confined to the default mode network^12,13^, a key system for self-referential processing and social cognition^21,22^. Changes in this network have been hypothesized to sustain the parents’ long-lasting ability to empathize with the infants and to reflect the profound restructuring of self-perception that parents often report^12,13^. However, our findings question the extent to which this network is the only one affected by pregnancy. The neuroanatomic location of our results indicates that the rest of the large-scale functional brain networks also undergo significant cortical reductions during pregnancy. However, unlike the reductions in other networks, those that fall into default mode and frontoparietal networks show below-expected volume increases in the early postpartum period. These findings suggest that whereas decreases in most networks, especially attentional ones, are likely to readjust or recover in the early postpartum period, those affecting higher-order cognitive networks, including the default mode network, may be traceable beyond that period. Indeed, cortical reductions in default mode regions have been detected at 1 (ref. ^13^), 2 (ref. ^12^) and 6 years postpartum^23^.

Another major milestone in the field is to elucidate which neuroplastic processes underlie the large neuroanatomic changes detected by MRI. Non-human mammal studies indicate that the parental transition comprises multiscale changes in the morphology of dendritic spines and branches and the proliferation of neurons and glial cells, altogether reflecting a ‘fine-tuning’ of the brain^2^. Although the transition to motherhood in human mothers may also involve changes at the molecular and cell morphology levels, these might contribute very little to the MRI signal^24^. Instead, it is more likely that the large changes in the brain observed by MRI in the current study reflect substantial neuroplasticity changes impacting entire cellular populations. Among the different types of brain cells, microglia, the innate immune cells of the brain, have been shown to follow a dynamic trajectory similar to that reported in the current study. According to rodent models, the density of microglia follows a trajectory of decreases during pregnancy that reverts across the postpartum period^25^. The functionality of reduced microglial proliferation across pregnancy and its recovery after parturition is yet to be elucidated. It is possible that decreased microglia during pregnancy mimics the anti-inflammatory state of the peripheral immune system^26^, thus reducing immune surveillance and inflammation in the brain. In addition, given the well-described role of activated microglia in pain hypersensitivity^27^, microglia depletion could function as a pain dampener, thus preparing the maternal brain for the extreme event of labor.

This study sheds light on the previously unexplored process of childbirth-related neuroplasticity. Our data show that women who had pre-labor C-sections presented greater cortical increases from late pregnancy to early postpartum than those who initiated labor. Pregnancy-induced brain changes have been widely argued to prepare the brain for motherhood^12,13^. However, brain adaptations in mothers may also be necessary for the process of childbirth itself^28^. Labor, which comprises the phases of dilatation, expulsion and placental stage, is a unique event at the hormonal, immunological and physiological level^29^. During the first stage of labor, that is, dilatation, pro-inflammatory signals are released. Combined with the effects of estrogens, prostaglandins and oxytocin, they trigger uterine contractions, cervical dilation, cervical effacement and the rupture of fetal membranes^30^. Although we cannot test whether pregnancy-induced brain adaptations prepare the mother’s brain for childbirth, our findings suggest that the extreme cascade of immune and endocrine adaptations of labor may also induce neuroplasticity. It is possible that mothers experiencing at least the first stage of labor may undergo further cortical reductions, reaching a lower cortical volume before reversing the trajectory (Supplementary Fig. 9a). An alternative explanation could be that mothers who did not initiate labor have a faster neural recovery. Although we did not find evidence of a steeper association between cortical increases and the percentage of postpartum time in the scheduled C-section group, mothers who undergo a scheduled C-section may potentially reach a recovery plateau before the postpartum session, preventing us from completely dismissing the faster recovery hypothesis (Supplementary Fig. 9b). These results emphasize the necessity to further investigate the neural events of childbirth and do not imply that scheduled C-section is detrimental to maternal neuroplasticity.

In a previous study, we found associations between changes in the cerebral cortex during pregnancy and maternal attachment metrics^12^. Our longitudinal findings, consistent with previous meta-analyses^31,32^, evidence the impact of the childbirth experience on postpartum wellbeing in terms of stress and sleep quality and the effect of this, in turn, on postnatal depression and maternal attachment. However, we did not detect significant associations between changes in neuropsychological scores and cortical changes. This apparent inconsistency may be because of differences in the experimental design. In a previous study, we tracked changes during pregnancy^12^, whereas in this one, we are tracking changes during the peripartum. This suggests that the cortical adaptations previously associated with maternal attachment might initiate before peripartum. In fact, during late pregnancy, mothers already scored high on maternal attachment, and their brain anatomy was already different from that of nulliparous women. Thus, it is possible that our peripartum time window may not be capturing the association between cortical changes and neuropsychological variables.

This study tracked the cortical changes of pregnant and non-pregnant women with the largest longitudinal sample of mothers ever explored by neuroimaging. Still, some caveats need to be considered when interpreting the results.

Including late pregnancy and early postpartum scans allowed a closer look at women’s brain changes around the peripartum, which remained a blindspot in neuroscience. However, the study lacks a pre-conception measure that serves as a baseline for neural and neuropsychological changes. Future studies should obtain brain images before conception and at multiple time points during pregnancy and postpartum to delineate the neural and neuropsychological adaptations that accompany pregnancy and the postpartum period, as well as the potential associations between them. Likewise, here we focused on characterizing changes at the cortical level. These data should be complemented with studies that delineate subcortical regions relevant to the human maternal brain^33^, using image acquisition parameters and image processing methods optimized for this purpose.

Another limitation is that we did not collect bio-markers of the endocrine and immune systems. Pregnancy hormones are widely recognized to play a critical role in regulating the maternal brain and behavior in rodents^34,35^, and have been previously associated with cortical changes in human mothers^13^. In addition to endocrine factors, the immune climate of pregnancy represents another potential mediating factor that warrants further attention. Among brain immune factors, the application of MRI spectroscopy^36^ and diffusion-based markers^37^ optimized to target microglial activity would help to confirm the contribution of microglia in the human maternal brain. Additionally, studying the cross-talk between glial markers and peripheral immune cells is a promising avenue for maternal brain research.

Here, we analyzed the effects of childbirth on the maternal brain by identifying differences between mothers who initiated labor (including those who had an emergency C-section after initiating labor) and mothers who had a scheduled C-section. Although the present neuroimaging study follows longitudinally the largest sample of mothers to date, mothers who gave birth by planned C-section accounted for only 10% of the sample. Thus, these findings should be confirmed with a larger sample of mothers who undergo scheduled C-sections. In addition, future studies should collect a broader battery of obstetric variables that allow a more comprehensive characterization of the parturition process.

Finally, it is important to note that literature on the maternal brain largely relies on Western samples of highly educated mothers with a medium–high socio-economic status. The generalizability of the present findings requires examining more diverse samples of mothers in terms of education, socio-economic status, race/ethnicity and cultural backgrounds.

To conclude, this work suggests a dynamic trajectory of cortical decreases during pregnancy that attenuates in the postpartum, at a different rate depending on the brain network and childbirth type. Altogether, these findings position the perinatal period as a sensitive and vulnerable time for women’s neuroplasticity that deserves protection and further study.

## Methods

This research complies with all the ethical regulations of the Instituto de Investigación Sanitaria del Hospital Gregorio Marañón and the Universitat Autònoma de Barcelona. We used a case-control longitudinal study assessing first-time mothers (*n* = 110) during late pregnancy and early postpartum and a control group of nulliparous women (*n* = 34). The main results were tested in an independent longitudinal sample formed of 29 mothers and 24 controls. Participants were assigned to either the experimental or control groups based on their pregnancy status. The type of delivery was determined by medical decisions and the participants’ choice. No statistical methods were used to pre-determine sample sizes, but our sample sizes were similar to or larger than those reported in previous publications^12,13^. Owing to the objectives and design of the study, data collection and analysis were not performed blind to the conditions of the experiments. We compensated participants with €50 per session for their time and commute. The flow of participants is shown in a diagram in Fig. 1, and demographic and gestational information is presented in Supplementary Table 1. Below, we describe the methodology of the main dataset, followed by that of the replication sample.

### Main dataset

#### Design and participants

A total of 110 first-time mothers aged 24–43 years old (mean ± s.d., 33.12 ± 3.98 years old) were assessed at the end of the third trimester of their first pregnancy (Prg; mean ± s.d., 36.23 ± 0.96 weeks) and during the first month postpartum (Post; mean ± s.d., 22 ± 8 days). As a control group, 34 age-matched nulliparous women (mean ± s.d., 33.32 ± 4.56 years old) were assessed at an equivalent time interval (mean ± s.d., 44 ± 10 days).

Participant recruitment was performed through word-of-mouth and the research group’s social media channels (Instagram, @neuro.maternal; X, formerly Twitter: @neuromaternal). Candidates who expressed interest in participating contacted us by email.

Exclusion criteria included an estimated intelligence quotient below 80 (estimated by Wechsler Adult Intelligence Scale (WAIS-IV) Digit Span subtest scores^38^), previous pregnancies beyond the first trimester, being a foster parent, gestating twins, past or current neurological disorders and past or current psychiatric conditions as assessed by the MINI International Neuropsychiatric Interview^39^. Among the 144 participants of the main sample, seven presented anxiety symptoms that did not meet the MINI diagnostic criteria (four of them pertained to the mothers’ group).

#### Data acquisition protocol

A week before scheduling the first MRI session, participants completed a series of self-reported questions administered by Qualtrics. These included questions about socio-demographic, medical and health information as well as questionnaires assessing sleep quality (PSQI^40^) and perceived stress (PSS^41^). In the mothers’ group, the survey also included measures of antenatal mother-to-infant attachment (Maternal Antenatal Attachment Scale, MAAS^42^), depression symptoms (Edinburgh Depression Antenatal Scale, EDAS^43^) and pregnancy anxiety (Pregnancy Anxiety Scale, PRAS^44^).

After the first MRI session was completed, pregnant women were asked to notify us as soon as they gave birth. Once they informed us, the second MRI session was scheduled. Before the second session, all participants were sent a Qualtrics link that included PSQI^40^ and PSS^41^. For the mothers’ group, we also collected obstetric and parenting information as well as measures of postnatal depression symptoms (Edinburgh Depression Postnatal Scale, EDPS^45^), maternal stress about their parenting role (Maternal Stress Scale, MSS^46^), postnatal mother-to-infant attachment (Maternal Postnatal Attachment Scale, MPAS^47^) and the birth experience (Birth Experience Questionnaire, BEQ^48^). The MRI sessions of the control group were scheduled to match the average intersession time of the mothers’ group.

Mothers and controls did not differ in terms of age (*t*_142_ = −0.24, *P* = 0.81), intersession time (*t*_142_ = −1.43, *P* = 0.15), WAIS-IV digits (*t*_141_ = 0.24, *P* = 0.33) or educational level (*χ*^2^_2,144_ = 1.52, *P* = 0.47). Among mothers, 11 women had a scheduled C-section (mean ± s.d. age, 32.11 ± 3.65 years), 12 had an emergency C-section (mean ± s.d. age, 34.13 ± 4.25 years) and 87 had a vaginal delivery (mean ± s.d. age, 33.11 ± 3.99 years). Thus, 99 women experienced the physiological events of the first stage of labor (‘labor mothers’; mean ± s.d. age, 33.23 ± 4.02 years). The remaining 11 women underwent scheduled C-sections (nine cases of breech position, one because of a previous myomectomy and one by choice) and thus were classified as not experiencing the physiological events of labor (‘pre-labor mothers’). Labor mothers did not differ from pre-labor mothers in age (*t*_108_ = 0.88, *P* = 0.38), intersession time (*t*_108_ = −0.45, *P* = 0.68), gestation weeks at Prg (*t*_10.91_ = 0.88, *P* = 0.40), WAIS-IV digits (*t*_108_ = 0.25, *P* = 0.80) or educational level (*χ*^2^_2,110_ = 0.73, *P* = 0.69). There were differences in gestational weeks at childbirth (*t*_108_ = 4.48, *P* < 0.0001), postpartum time (*t*_108_ = −3.19, *P* = 0.002) and percentage of postpartum time between sessions (*t*_109_ = −3.82, *P* = 0.0002). Further details regarding demographic data are depicted in Supplementary Table 1.

The described design and procedures complied with data protection regulations and were approved by the ethical committee of the Instituto de Investigación Sanitaria del Hospital Gregorio Marañón according to the Declaration of Helsinki guidelines. All participants signed a consent form before participating in the study.

#### MRI data acquisition

We acquired a three-dimensional T1-weighted image for each participant and session (Prg and Post) on a Siemens MAGNETOM Vida with a Head-and-Neck 20 channel coil, located at Hospital Beata Maria Ana (Madrid, Spain). We used a magnetization prepared rapid gradient-echo (MPRAGE) sequence in sagittal orientation with the following parameters: voxel size, 0.9375 × 0.9375 × 1 mm^3^; field of view, 240 × 240 × 176 mm^3^; echo time, 44 ms; repetition time, 9.8/2300 ms; inversion time, 900 ms; flip angle, 8^∘^; GRAPPA acceleration factor, 2; percent sampling, 80%; acquisition time, 265 s. We performed a visual quality check on site and repeated the acquisition when artifacts were detected. None of the participants needed to be excluded because of low data quality.

#### Image processing

To process the images, we used the *recon-all* longitudinal stream in FreeSurfer version 7.1.1 (ref. ^49^). First, we processed the individual Prg and Post brain scans of mothers and controls cross-sectionally (Supplementary Fig. 10). This cross-sectional pipeline models the outer (pial) and inner (white matter) cortical boundaries, yielding cortical volume, cortical thickness and white surface area vertex-wise maps for each participant and session independently. The pipeline also provides the estimated total intracranial volume and the Euler number, whose average across hemispheres is an excellent proxy for image quality^50^. Mean Euler number did not differ between mothers and controls at Prg (*t*_142_ = −0.56, *P* = 0.57) or Post sessions (*t*_142_ = −0.35, *P* = 0.73). Furthermore, there was no difference in estimated total intracranial volume between the two groups (*t*_142_ = 0.87, *P* = 0.39).

In addition, each participant’s Post brain scan was processed in relation to their Prg scan with a longitudinal workflow (Supplementary Fig. 11). This longitudinal workflow creates an unbiased template from the individual Prg and Post images. Each participant’s template served to initialize the reconstruction of the session-specific surfaces. The outputs are participant-specific cortical surfaces with the same number of vertices and faces in both sessions, thus improving the intra-participant precision of the metrics.

We analyzed MRI data both at the global and vertex-wise levels. For global metrics, we used the cortical maps in the subjects’ anatomical space to calculate total cortical volume, mean cortical thickness and total surface area. For vertex-wise analyses, we projected the subjects’ cortical maps onto the common space *fsaverage* and applied a 10 mm full-width-at-half-maximum Gaussian kernel filter for smoothing.

#### Statistical analyses

##### Linear mixed effects model

Data were analyzed using linear mixed effects (LME) models. For global analyses, we fitted separate LME models using total cortical volume, mean cortical thickness and total surface area as dependent variables. In all the models, we used group (mothers vs controls), session (Prg vs Post) and the group × session interaction as independent variables. To account for subject-specific differences, random intercepts were incorporated into the models. Wald *F*-tests were used to assess the two-tailed group differences during Prg and Post sessions, as well as the longitudinal changes given by the group × session interaction. In addition, we calculated signed effect sizes as partial eta squared (${\eta }_{p}^{2}$), considering the sign of the parameter associated with each contrast. As supplementary analyses, age, total intracranial volume and mean Euler number were included as fixed effects to control for these potentially confounding factors. Given that mothers and controls differed significantly in perceived stress and sleep quality (Supplementary Table 9), we fitted an additional model that included age, total intracranial volume and mean Euler number as well as perceived stress and sleep quality.

Additional LME models were fitted in the mothers’ group to test the effects of labor (‘labor’ vs ‘pre-labor’). As pre-labor mothers differed from labor mothers in terms of gestational weeks at childbirth and postpartum days (Supplementary Table 12), we fitted a model that included gestational weeks at childbirth and postpartum days, together with age, total intracranial volume and mean Euler number as confounding variables in a supplementary analysis. Post-hoc models were also fitted to disentangle the effects of vaginal delivery, emergency C-section and scheduled C-section.

For each of the above-mentioned three sets of independent variables (mothers vs controls, labor vs non-labor and vaginal delivery vs emergency C-section vs. scheduled C-section), we corrected *P* values using a false discovery rate (FDR) correction across contrasts and cortical metrics. We considered FDR-corrected *P* values below a threshold of 0.05 to be significant.

To localize the global differences within the brain, we fitted the same LME models (mothers vs controls and labor vs non-labor) at a vertex-wise level using the *fslmer* R library, which ports to the R language the original Matlab LME vertex-wise implementation^51^. For each model and contrast, we corrected vertex-wise *P* values using an FDR correction across hemispheres. We considered FDR-corrected *P* values below a threshold of 0.05 to be significant. Then, we obtained a list of the affected cluster regions based on the Desikan–Killiany atlas^52^.

Neuropsychological data were also analyzed using LME models, with group (mothers vs controls), session (Prg vs Post) and the group × session interaction as the independent variables. For those questionnaires that were only administered to the mothers group, session (Prg vs Post) was the only independent variable included in the model. For each set of independent variables, we corrected *P* values using FDR across all questionnaires and contrasts.

The normality of the distributions was ensured by examination of skewness and kurtosis. All variables were normally distributed. Equal variances were tested through *F*-tests for group and labor comparisons. Mothers and controls had unequal variances in Prg-to-Post sleep problems score and Post global cortical thickness variables. The rest of the variables had equal variances.

##### Spatial correspondence with large-scale functional networks

We computed the mean signed effect size in each of the seven large-scale functional networks described in a previous work^19^ and compared them with suitable null distributions to assess which networks showed significantly lower or higher spatial correspondence with the observed cortex differences. Null distributions were generated using spin-permutations (rotations) of the maps and then computing the mean values in each network (that remained unrotated)^53^ again. Specifically, we computed 1,000 uniformly distributed random rotations of the *fsaverage* vertex indices using the *spin-test* toolbox (https://github.com/spin-test/spin-test), which were then used to obtain the rotated maps and the null distributions. Finally, we computed *P* values for each map and network as the proportion of rotations that yielded higher or lower values than our original maps. We considered *P* values below a threshold of 0.05 to be significant.

##### Correlation analyses in the mothers’ group

For correlation analyses, we calculated the Prg-to-Post percentage of change for each global metric (cortical volume, cortical thickness and surface area) using the following formula: $\frac{(\rm{Post} - \rm{Prg})}{\rm{Prg}}{\times}100$. Notice that the percentage of change highly correlates with the difference of residuals between Post and Prg sessions when fitting an LME model including group (mothers vs controls) as an independent variable (see Supplementary Fig. 12). Two-tailed Pearson correlations were used to determine whether the mothers’ percentage of change in cortical metrics was associated with the percentage of postpartum time between the sessions, that is, the rate between postpartum time (days between the childbirth and the Post session) and the intersession time (days between MRI sessions). We considered *P* values surviving FDR correction with *q* = 0.05 as significant. The same Pearson correlations were also calculated differentiating labor from pre-labor mothers.

The association between neuropsychological variables as well as between these variables and the percentages of change in global cortical metrics were also tested by two-tailed Pearson correlations. For each of these correlation matrices, we considered *P* values surviving FDR correction with *q* = 0.05 as significant.

##### Software

Statistical analyses were performed in Rstudio (version 2022.02.3+294), under R version 4.2.1, with the following libraries: *fslmer* (version 0.0.0.9002) for LME models, including global, vertex-wise and neuropsychological data; and *stats* (version 4.2.1) for correlations and FDR correction^54^. Vertex-wise FDR-corrected maps were computed using FSL *fdr* function (version 6.0.5). The cluster locations of the vertex-wise results were obtained using FreeSurfer’s *mri_surfcluster* function (version 7.2.0). Spin tests were performed using the *spin-test* toolbox (https://github.com/spin-test/spin-test) and the following Python (version 3.9.7) packages: *nibabel* (version 3.2.1), *pandas* (version 1.5.3), *numpy* (version 1.21.5) and *scipy* (version 1.10.1).

Figures were plotted using *ggplot2* (version 3.3.6), *GGally* (version 2.1.2), *ggpubr* (version 0.4.0), ggbeeswarm (version 0.7.1) and *corrplot* (version 0.9.2) R libraries. The in-house Rscript also used the following libraries: dplyr (version 1.0.9), feather (version 0.3.5), moments (version 0.14.1), tidyr (version 1.2.0), stringr (version 1.4.0), reshape2 (version .8.9), gtsummary (version 1.6.1), gt (version 0.6.0), cowplot (version 1.1.1), knitr (version 1.39), kableExtra (version 1.3.4), latex2exp (version 0.9.6) and gridExtra (version 2.3). Vertex-wise analyses were plotted using the *nilearn* (version 0.9.1), *nibabel* (version 3.2.1), *seaborn* (version 0.12.2) and *matplotlib* (version 3.6.2) Python packages.

### Replication dataset

#### Design and participants

To test the reliability of our findings, we performed a replication study with an independent sample of 29 first-time mothers (mean ± s.d. age, 32.74 ± 3.76 years) and 24 age-matched nulliparous women (mean ± s.d. age, 31.49 ± 3.35 years) assessed at similar Prg (mean ± s.d., 34.32 ± 0.84 weeks) and Post time points (mean ± s.d., 33.1 ± 5.8 days). This independent sample was formed by different individuals whose brain images were acquired using a different MRI scanner located in a different city in Spain, and using slightly different image acquisition parameters (see ‘MRI data acquisitionsʼ).

Participant recruitment was performed through word-of-mouth and the research group’s social media channels. Candidates who expressed interest in participating contacted us by email.

Exclusion criteria included estimated intelligence quotient below 80 (estimated by WAIS Digit Span subtest scores^38^), previous pregnancies beyond the first trimester, being a foster parent, gestating twins, current or history of neurological conditions and current or history of psychiatric conditions as assessed by the MINI International Neuropsychiatric Interview^39^. Among the 53 participants of the replication sample, two mothers had a history of affective disorder co-occurring with anxiety disorder, four controls had a history of anxiety disorders and one control had a history of affective disorder co-occurring with anxiety disorder. Supplementary Table 1 shows the demographic and gestational information of the replication dataset.

#### Data acquisition protocol

The data evaluation protocol was equivalent to that described for the main dataset. Mothers and controls did not differ in terms of age (*t*_51_ = −1.27, *P* = 0.21), intersession time (*t*_51_ = 0.77, *P* = 0.45), WAIS-IV digits (*t*_49_ = 1.48, *P* = 0.15), mean Euler number at Prg (*t*_51_ = −0.99, *P* = 0.33) and Post (*t*_51_ = −0.75, *P* = 0.46) or educational level (*χ*^2^_2,53_ = 0.03, *P* = 0.87).

Among mothers, two women had a scheduled C-section, five had an emergency C-section and 22 had a vaginal delivery. Thus, 27 women experienced the physiological events of the first stage of labor (labor mothers). The described design and procedures were approved by the Ethics Committee on Human and Animal Experimentation at the Universitat Autònoma de Barcelona according to the Declaration of Helsinki guidelines. All participants signed a consent form before participating in the study.

#### MRI data acquisitions

We acquired a three-dimensional T1-weighted image for each participant and session (Prg and Post) on a Philips Ingenia CX system with a Head-and-Neck 32-channel coil, located at the Barcelona Beta Brain Research Center (Barcelona, Spain). We used a turbo field echo sequence in sagittal orientation and the following parameters: voxel size, 0.75 × 0.75 × 1 mm^3^; field of view, 240 × 240 × 180 mm^3^; echo time, 46 ms; repetition time, 9.9/2,300 ms; prepulse delay, 900 ms; flip angle, 8^∘^; acceleration factor, 1.9; percent sampling, 78%; acquisition time, 259 s. We performed a visual quality check on site and repeated the acquisition when artifacts were detected. None of the participants needed to be excluded because of low data quality.

#### Image processing and statistical analyses

Image processing and vertex-wise neuroanatomic statistical analyses were performed, mimicking those implemented in the main dataset, with the exception of the FreeSurfer update to version 7.2. Given the smaller sample size of the replication dataset, we did not test for differences in cortical changes as a function of labor. However, we depicted the distribution of the data as a function of labor in Supplementary Fig. 8. Normal distributions and equal variances were ensured through skewness and kurtosis and *F*-tests, respectively. All variables were normally distributed with the exception of postpartum days.

For vertex-wise data, we implemented a similar spin-test strategy as in ‘Spatial correspondence with large-scale functional networks’ to assess the spatial correspondence of the obtained signed effect size maps between the main and replication samples. We used Pearson’s correlation as the similarity metric and computed *P* values as the proportion of rotations that yielded equal or higher correlation coefficients.

### Reporting summary

Further information on research design is available in the Nature Portfolio Reporting Summary linked to this article.

## Online content

Any methods, additional references, Nature Portfolio reporting summaries, source data, extended data, supplementary information, acknowledgements, peer review information; details of author contributions and competing interests; and statements of data and code availability are available at 10.1038/s41593-023-01513-2.

### Supplementary information

Supplementary Information Supplementary Tables 1–12 and Figs. 1–12.

Reporting Summary

## Data Availability

The datasets including the global cortical metrics, demographic information, obstetric data and neuropsychological information generated and analyzed in the current study are available on the GitHub repository (https://github.com/neuromaternal/peripartum_neuroplasticity). Effect sizes and significance vertex-wise maps reported in the manuscript are also available there. Additionally, we have also uploaded 3D interactive maps of the studied contrasts for the readers to explore the data. All data and code necessary to replicate and extend our findings are available in the repository. The transfer of the raw and processed MRI images of the study participants requires additional data treatment agreement including the purpose of the use and thus are only available upon reasonable request to the corresponding author.

## Code availability

Image processing and vertex-wise statistical analyses of the neuroimaging data are based on pipelines integrated within the software referenced in the Methods section. Custom code generated for additional statistical analyses and figure representations is available on the GitHub repository (https://github.com/neuromaternal/peripartum_neuroplasticity), along with the necessary datasets to replicate them.
