# Supplementary material for: Women’s neuroplasticity during gestation, childbirth and postpartum
Source: Nat Neurosci. 2024 Jan 5;27(2):319–27. doi: 10.1038/s41593-023-01513-2 (PMC10849958; doi:10.1038/s41593-023-01513-2)
Supplement: Supplementary file 1 — Supplementary Tables 1–12 and Figs. 1–12. [file 41593_2023_1513_MOESM1_ESM.pdf]

nature neuroscience

Article https://doi.org/10.1038/s41593-023-01513-2

Women's neuroplasticity during gestation, childbirth and postpartum

In the format provided by the authors and unedited

Supplementary Material: Women's neuroplasticity during gestation, childbirth, and postpartum

Table SM 1 Demographics of the main and replication datasets.

	Main Dataset		Replication Dataset	
**Characteristic**	**Controls**, N = 34	**Mothers**, N = 110	**Controls**, N = 24	**Mothers**, N = 29
**Age at pregnancy session [years]**				
Mean (SD)	33.32 (4.56)	33.12 (3.98)	31.49 (3.35)	32.74 (3.76)
Minimum-Maximum	25.41–45.98	24.10–43.79	25.04–39.38	26.12–38.50
**Gestational weeks at pregnancy session [weeks]**				
Mean (SD)		36.23 (0.96)		34.32 (0.84)
Minimum-Maximum		33.14–38.71		33.57–36.43
**Gestational weeks at childbirth [weeks]**				
Mean (SD)		39.67 (1.06)		39.76 (1.51)
Minimum-Maximum		37.10–41.50		36.29–42.43
**Type of parturition [nos. of participants (%)]**				
Vaginal		87 (79%)		22 (76%)
Emergency C-section		12 (11%)		5 (17%)
Scheduled C-section		11 (10%)		2 (6.9%)
**Postpartum time [days]**				
Mean (SD)		21.68 (8.05)		33.07 (5.84)
Median (IQR)		20 (16, 25.75)		31.00 (30, 34)
Minimum-Maximum		7–53		26–55
**Time between sessions [days]**				
Mean (SD)	43.97 (10.11)	47.15 (11.67)	73.54 (11.74)	71.16 (10.71)
Minimum-Maximum	31–70	27–90	57–98	51–87
**Percentage of postpartum time between sessions**				
Mean (SD)		46.43 (13.79)		47.61 (11.19)
Minimum-Maximum		12.73–82.14		33.33–73.68
**Education [nos. of participants (%)]**				
Primary	0 (0%)	4 (3.6%)	0 (0%)	0 (0%)
Secondary	6 (17.6%)	15 (13.6%)	2 (8.3%)	1 (3.4%)
Higher	28 (82.4%)	91 (82.7%)	22 (91.7%)	28 (96.6%)
**WAIS-IV Digits**				
Mean (SD)	28.67 (5.39)	27.71 (4.76)	29.41 (4.45)	27.45 (4.85)
Minimum-Maximum	18–44	16–44	23–40	18–42
**Mean *Euler* number at pregnancy session**				
Mean (SD)	−17.21 (5.76)	−16.53 (6.27)	−12.92 (6.39)	−11.03 (7.31)
Minimum-Maximum	−31–−6	−34–−6	−26–−1	−32–−2
**Mean *Euler* number at postpartum session**				
Mean (SD)	−15.91 (5.59)	−15.50 (6.13)	−12.50 (7.63)	−11.14 (5.60)
Minimum-Maximum	−26–−5	−39–−5	−33–−1	−26–−1

Datasets did not differ neither in age (t(195)=1.55; p=0.12), WAIS-IV digits (t(192)=−0.46; p=0.65), nor educational level (χ^2(2,197)=4.62; p=0.10), but differed in intersession days (t(195)=−14.22; p=5.75e-32). In mothers, no differences were found in gestational weeks at delivery (t(35.52)=−0.33; p=0.75), percentage of postpartum time between sessions (t(137)=−0.42; p=0.67), or type of parturition (χ^2(2,139)=1.02; p=0.60). However, mothers' differed in gestational weeks at pregnancy session (t(137)=9.80; p=1.72e-17 and postpartum time (W=381.5; p=2.87e-10). P-values below the threshold of 0.0001 are reported in exponential notation.
There were no differences between mothers and nulliparous women ("Controls") in terms of age, time between sessions, mean *Euler* number at both sessions, or education in any of the datasets. Details of the within-dataset comparisons can be found in "Methods"—Data acquisition protocol (see Section 4.1.2).
The main sample size was 144 (110 mothers and 34 controls) in all variables except for the WAIS-IV, which had missing data from one control. The replication sample size was 53 (29 mothers and 24 controls) in all variables except for the WAIS-IV, which had missing data from two controls.
All variables were normally distributed except for the mothers' postpartum time of the replication dataset. For this variable, the median and interquartile ranges were also reported. All variables had equal variances between datasets except for the mothers' gestational weeks at childbirth. Therefore, two-tailed two-sample student T-tests were employed across variables except for between-dataset comparisons of mothers' postpartum time (two-tailed Wilcoxon non-parametric test) and mothers' gestational weeks at childbirth (two-tailed *Welch*'s T-test). No multiple comparisons corrections were applied. Abbreviations: IQR=interquartile range, SD= standard deviation, and WAIS-IV= Wechsler Adult Intelligence Scale.

Table SM 2 Descriptives and group comparison statistics of the global differences in cortical metrics at Pregnancy session ("Prg"), Postpartum session ("Post"), and "Prg-to-Post" in mothers and nulliparous women ("Controls").

	**Controls, N=34**		**Mothers, N=110**		**Group differences**		
**Metric/Statistic**	**Prg**	**Post**	**Prg**	**Post**	**Prg**	**Post**	**Prg-to-Post**
**Cortical Volume**							
Mean	469.19	468.00	447.86	450.94			
Standard deviation	33.70	33.59	29.51	29.73			
F-statistic					12.67	8.10	12.94
Degrees of freedom					1, 144.80	1, 144.80	1, 142.00
Uncorrected p-value					0.0005*	0.0051*	0.0004*
Signed effect size					−0.0804	−0.053	0.0835
**Cortical Thickness**							
Mean	2.47	2.47	2.41	2.42			
Standard deviation	0.08	0.09	0.07	0.06			
F-statistic					20.24	10.43	12.43
Degrees of freedom					1, 151.47	1, 151.47	1, 142.00
Uncorrected p-value					1.35e-05*	0.0015*	0.0006*
Signed effect size					−0.1179	−0.0644	0.0805
**Surface Area**							
Mean	1683.31	1681.19	1655.83	1658.48			
Standard deviation	114.77	113.95	110.01	110.23			
F-statistic					1.59	1.09	7.10
Degrees of freedom					1, 142.48	1, 142.48	1, 142.00
Uncorrected p-value					0.2087	0.2985	0.0086*
Signed effect size					−0.0111	−0.0076	0.0476

The adjusted linear mixed effect model corresponds to $CorticalMetric \sim 1 + Group + Session + Group \times Session + (1 \mid Participant)$. Degrees of freedom were obtained using Satterthwaite's approximation. P-values below the threshold of 0.0001 are reported in exponential notation. Multiple comparisons were applied across metrics and contrasts. Asterisks indicate a False Discovery Rate-corrected p-value<0.05. Effect sizes were calculated as the signed partial eta squared (η_p^2) associated with the correspondent one-tailed *Wald* F-tests.

Table SM 3 Regions of cortical volume and thickness differences in mothers (N=110) compared to nulliparous women ("Controls", N=34) at late pregnancy.

Metric	Hemisphere	#	Region (*aparc*)	Size (cm^2)	MNI coordinates (x, y, z)	Vertex p min	FDR-corrected p min
Cortical Volume	Left	1	frontalpole	10.25	(-8.0, 64.4, -2.3)	258	0.0073
		2	temporalpole	2.14	(-41.8, 11.3, -33.8)	43921	0.0124
		3	superiorfrontal	2.01	(-8.0, 49.9, 23.5)	93006	0.0086
		4	superiortemporal	1.16	(-48.5, 3.0, -17.5)	69820	0.0309
		5	lateralorbitofrontal	1.02	(-35.0, 27.3, -10.1)	124677	0.0191
	Right	1	paracentral	5.66	(4.1, -39.3, 66.4)	712	0.0076
		2	postcentral	4.59	(59.2, -10.6, 35.1)	2099	0.0089
		3	superiorfrontal	4.23	(17.3, 64.2, 1.8)	51218	0.0098
		4	precentral	3.09	(51.9, -3.9, 45.9)	11292	0.0140
		5	superiorfrontal	2.88	(8.1, 55.5, 26.5)	743	0.0182
		6	postcentral	2.53	(40.7, -29.7, 63.7)	1580	0.0076
		7	superiorparietal	2.44	(27.3, -57.8, 46.0)	21252	0.0175
		8	superiortemporal	1.87	(52.4, 7.7, -15.6)	163301	0.0145
		9	middletemporal	1.48	(64.9, -23.8, -13.3)	48327	0.0311
		10	postcentral	1.24	(49.8, -21.5, 54.3)	26808	0.0361
		11	precentral	1.21	(58.3, 5.6, 28.6)	2145	0.0089
		12	inferiortemporal	1.04	(51.9, -46.2, -18.5)	70312	0.0200
Cortical Thickness	Left	1	superiorfrontal	43.44	(-7.1, 42.8, 37.6)	250	0.0003
		2	paracentral	14.50	(-16.1, -36.7, 49.9)	307	0.0003
		3	superiorparietal	14.32	(-21.2, -61.1, 41.9)	2322	0.0003
		4	inferiortemporal	13.77	(-54.0, -40.6, -24.1)	75473	0.0033
		5	parsopercularis	12.09	(-50.1, 13.7, 15.4)	9198	0.0033
		6	precentral	11.26	(-25.3, -13.0, 53.2)	76862	0.0032
		7	middletemporal	9.22	(-55.9, -14.6, -19.6)	127574	0.0004
		8	superiortemporal	8.52	(-47.3, -6.9, -14.7)	18726	0.0003
		9	postcentral	6.44	(-29.4, -34.9, 59.0)	133249	0.0012
		10	lateralorbitofrontal	5.98	(-15.4, 24.6, -21.9)	54251	0.0028
		11	inferiorparietal	4.94	(-35.2, -57.9, 37.1)	78230	0.0062
		12	superiortemporal	4.05	(-49.4, -32.7, 5.6)	43471	0.0009
		13	lingual	3.31	(-28.4, -64.2, -5.6)	41733	0.0068
		14	precentral	2.67	(-38.3, 4.1, 13.0)	35172	0.0097
		15	lateralorbitofrontal	2.32	(-36.6, 27.0, -10.8)	60530	0.0016
		16	inferiorparietal	2.19	(-36.5, -80.3, 11.6)	68916	0.0025
		17	postcentral	2.07	(-46.2, -25.9, 38.1)	32842	0.0137
		18	rostralmiddlefrontal	2.06	(-40.0, 26.3, 16.3)	125063	0.0130
		19	precuneus	1.97	(-8.1, -53.6, 60.0)	2857	0.0092
		20	middletemporal	1.92	(-58.0, -30.2, -11.9)	148630	0.0032
		21	precuneus	1.84	(-21.3, -61.9, 16.9)	15300	0.0094
		22	rostralmiddlefrontal	1.79	(-19.6, 48.8, 30.7)	56286	0.0079
		23	supramarginal	1.69	(-56.8, -46.0, 35.9)	155961	0.0152
		24	inferiorparietal	1.69	(-37.4, -57.3, 21.0)	31250	0.0171
	Right	1	superiorfrontal	28.02	(7.6, 26.0, 56.8)	3516	0.0003
		2	inferiorparietal	20.48	(53.1, -51.5, 40.4)	6030	0.0009
		3	parsopercularis	17.81	(37.8, 8.3, 22.7)	2650	0.0020
		4	superiorparietal	14.59	(24.4, -58.8, 48.4)	1240	0.0003
		5	insula	12.00	(30.1, 20.8, 6.6)	154239	0.0005
		6	posteriorcingulate	10.55	(9.3, -8.3, 40.9)	146593	0.0014
		7	inferiorparietal	6.42	(44.1, -62.4, 24.1)	87630	0.0012
		8	superiortemporal	4.73	(54.9, 5.4, -13.4)	40787	0.0135
		9	middletemporal	4.13	(55.8, -4.8, -29.3)	163636	0.0103
		10	rostralmiddlefrontal	3.58	(37.6, 51.4, -3.8)	145877	0.0056
		11	middletemporal	3.28	(64.8, -19.8, -15.7)	3952	0.0060
		12	postcentral	3.11	(40.2, -31.0, 64.1)	24896	0.0188
		13	rostralmiddlefrontal	3.03	(46.2, 27.5, 31.0)	4686	0.0116
		14	precuneus	2.78	(7.6, -56.5, 45.3)	63625	0.0051
		15	insula	2.58	(35.3, -15.5, 19.7)	57559	0.0084
		16	superiorparietal	2.34	(14.4, -55.6, 59.4)	123732	0.0057
		17	middletemporal	2.09	(63.1, -47.6, -2.8)	62996	0.0038
		18	lingual	1.83	(26.7, -55.4, 3.3)	21198	0.0013
		19	superiorfrontal	1.74	(23.5, 12.0, 45.5)	105570	0.0043
		20	bankssts	1.73	(44.8, -40.9, 7.2)	160189	0.0035
		21	fusiform	1.37	(36.4, -12.5, -32.9)	24356	0.0136
		22	rostralmiddlefrontal	1.19	(21.3, 61.3, 3.8)	73396	0.0257
		23	rostralmiddlefrontal	1.10	(22.3, 51.5, -12.4)	29201	0.0148
		24	lateraloccipital	1.06	(47.0, -75.6, 9.6)	87741	0.0254

Vertex-wise statistical analyses of significant group differences in left and right cortical metrics using the linear mixed effect model: $Cortical\ Metric \sim 1 + Group + Session + Group \times Session + (1 \mid Participant)$. Clusters were obtained through the FreeSurfer *mri_surfcluster* function using the False Discovery Rate (FDR) corrected p-value maps from the one-tailed *Wald* F-tests. We used an α level of 5% and a minimum surface area of 1 cm^2. *Region* corresponds to the labels of the *Desikan-Killiany* atlas [52] , *size* corresponds to the surface area of the significant clusters while *MNI coordinates*, *vertex p min*, and *FDR-corrected p min* correspond to the peak with minimum p-value.

Table SM 4 Regions of cortical volume and thickness differences in mothers (N=110) compared to nulliparous women ("Controls", N=34) at early postpartum.

**Metric**	**Hemisphere**	#	**Region** (*aparc*)	**Size** (cm^2)	**MNI coordinates** (x, y, z)	**Vertex** p min	**FDR-corrected** p min
**Cortical Volume**	Left	1	frontalpole	3.66	(-8.0, 64.4, -2.3)	258	0.0043
		2	superiorfrontal	1.73	(-15.1, 61.3, 12.1)	92611	0.0157
		3	temporalpole	1.12	(-41.5, 11.5, -34.5)	143481	0.0258
	Right	1	postcentral	3.70	(59.2, -10.6, 35.1)	2099	0.0180
		2	superiorfrontal	2.74	(16.6, 64.6, 1.3)	1630	0.0225
		3	superiorfrontal	2.48	(9.4, 59.0, 26.1)	744	0.0249
		4	paracentral	2.26	(4.1, -39.3, 66.4)	712	0.0249
		5	postcentral	1.14	(39.6, -29.9, 63.9)	16528	0.0244
**Cortical Thickness**	Left	1	superiorfrontal	5.44	(-7.1, 42.8, 37.6)	250	0.0074
		2	paracentral	3.54	(-12.7, -36.5, 53.5)	2106	0.0074
		3	lateralorbitofrontal	3.25	(-15.5, 23.9, -21.8)	23335	0.0076
		4	superiorparietal	2.50	(-21.2, -61.1, 41.9)	2322	0.0074
		5	isthmuscingulate	2.02	(-13.5, -43.0, 32.4)	1296	0.0074
		6	superiorfrontal	1.65	(-12.1, 49.7, 7.3)	153161	0.0170
		7	inferiortemporal	1.45	(-53.4, -44.4, -23.2)	101935	0.0257
		8	superiortemporal	1.42	(-52.7, 7.2, -14.4)	89967	0.0260
		9	superiorfrontal	1.26	(-13.6, 31.6, 24.0)	134053	0.0077
		10	rostralmiddlefrontal	1.19	(-45.2, 26.1, 31.7)	80443	0.0252
		11	posteriorcingulate	1.09	(-4.1, -7.1, 36.7)	18619	0.0074
		12	postcentral	1.07	(-30.0, -35.0, 59.2)	66999	0.0205
		13	precentral	1.04	(-36.6, 3.2, 24.9)	66558	0.0136
		14	inferiorparietal	1.00	(-34.9, -59.0, 37.6)	147105	0.0229
	Right	1	superiorfrontal	8.37	(7.3, 29.0, 55.8)	1535	0.0074
		2	inferiorparietal	6.49	(51.5, -50.0, 40.0)	310	0.0074
		3	postcentral	4.64	(57.5, -15.4, 32.3)	61947	0.0124
		4	rostralmiddlefrontal	1.71	(46.3, 26.6, 31.5)	124133	0.0236
		5	superiorparietal	1.49	(24.4, -58.8, 48.4)	1240	0.0074
		6	inferiorparietal	1.16	(34.1, -72.2, 31.2)	147534	0.0299

Vertex-wise statistical analyses of significant group differences in left and right cortical metrics using the linear mixed effect model: $Cortical\,Metric \sim 1 + Group + Session + Group \times Session + (1 \mid Participant)$. Clusters were obtained through the FreeSurfer *mri_surfcluster* function using the False Discovery Rate (FDR) corrected p-value maps from the one-tailed *Wald* F-tests. We used an α level of 5% and a minimum surface area of 1 cm^2. *Region* corresponds to the labels of the *Desikan-Killiany* atlas [52] , *size* corresponds to the surface area of the significant clusters while *MNI coordinates*, *vertex p min*, and *FDR-corrected p min* correspond to the peak with minimum p-value.

Table SM 5 Regions of cortical volume, thickness, and surface area differences in mothers (N=110) compared to nulliparous women ("Controls", N=34) from Pregnancy-to-Postpartum.

**Metric**	**Hemisphere**	#	**Region** (*aparc*)	**Size** (cm^2)	**MNI coordinates** (x, y, z)	**Vertex** p min	**FDR-corrected** p min
Cortical Volume	Left	1	superiortemporal	30.28	(-47.2, -2.8, -16.9)	8503	<0.0001
		2	superiorparietal	22.16	(-32.6, -34.9, 39.2)	7500	0.0004
		3	inferiortemporal	20.44	(-45.2, -62.2, -0.6)	156733	0.0007
		4	precentral	17.41	(-54.9, 5.3, 4.0)	110488	0.0009
		5	lateraloccipital	12.73	(-17.0, -98.8, 6.7)	39222	0.0010
		6	isthmuscingulate	10.45	(-11.9, -46.1, 30.1)	69273	0.0037
		7	superiorfrontal	9.54	(-20.8, -6.5, 51.1)	17950	0.0022
		8	superiorfrontal	7.32	(-10.1, 6.0, 44.2)	20190	0.0035
		9	precuneus	5.97	(-17.6, -62.8, 26.9)	57553	0.0006
		10	superiorparietal	4.82	(-24.9, -67.9, 25.0)	51996	0.0027
		11	insula	4.30	(-28.5, 18.4, -5.7)	44284	0.0032
		12	lateraloccipital	3.88	(-23.2, -85.5, -12.0)	79744	0.0053
		13	pericalcarine	2.34	(-14.1, -82.2, 9.5)	143877	0.0051
		14	supramarginal	2.11	(-54.3, -49.8, 31.8)	7314	0.0082
		15	inferiortemporal	1.95	(-49.1, -36.9, -24.4)	128012	0.0225
		16	precentral	1.80	(-38.8, -16.0, 58.5)	97947	0.0052
		17	lingual	1.26	(-6.3, -74.8, -0.4)	118394	0.0105
		18	superiorparietal	1.25	(-35.3, -49.8, 60.7)	1042	0.0171
		19	postcentral	1.04	(-41.8, -19.7, 18.0)	65823	0.0072
	Right	1	insula	35.81	(37.8, -4.1, 0.5)	197	<0.0001
		2	precuneus	25.39	(20.0, -60.7, 26.5)	61426	0.0002
		3	middletemporal	23.65	(53.7, -58.1, 5.9)	34271	0.0010
		4	paracentral	19.35	(6.1, -9.6, 55.3)	1027	<0.0001
		5	superiortemporal	16.21	(53.1, -19.2, -3.9)	91781	<0.0001
		6	precentral	10.90	(37.4, -8.4, 57.3)	23462	0.0002
		7	precentral	3.40	(57.5, 5.4, 30.1)	60277	0.0041
		8	lateraloccipital	3.24	(17.3, -100.1, 9.3)	111558	0.0077
		9	superiorparietal	2.87	(33.5, -44.6, 49.8)	12359	0.0028
		10	superiorfrontal	2.57	(10.4, 33.5, 30.6)	149309	0.0013
		11	fusiform	2.14	(42.5, -47.1, -13.4)	32049	0.0045
		12	precuneus	1.98	(6.4, -48.3, 51.7)	123816	0.0023
		13	lateraloccipital	1.84	(17.1, -87.6, -7.5)	137054	0.0053
		14	lateralorbitofrontal	1.70	(33.5, 25.8, -10.5)	37714	0.0074
		15	lingual	1.62	(28.7, -62.1, -4.4)	75473	0.0059
		16	postcentral	1.53	(44.7, -29.5, 60.2)	117000	0.0106
		17	precuneus	1.30	(9.5, -58.4, 41.9)	71263	0.0064
		18	precuneus	1.20	(7.7, -69.5, 49.9)	49140	0.0151
		19	inferiortemporal	1.14	(46.0, -3.7, -32.2)	48089	0.0107
Cortical Thickness	Left	1	superiortemporal	2.79	(-48.6, -0.5, -15.8)	433	0.0143
		2	superiortemporal	2.01	(-49.9, -29.9, 3.0)	100998	0.0280
		3	lateraloccipital	1.27	(-26.7, -83.4, -13.9)	107756	0.0285
		4	superiorfrontal	1.18	(-20.8, -7.2, 52.5)	25746	0.0203
	Right	1	precentral	6.34	(37.9, -7.6, 55.6)	875	0.0203
		2	paracentral	4.42	(14.5, -19.8, 44.8)	8747	0.0143
		3	superiorparietal	1.83	(19.5, -80.3, 40.1)	115772	0.0230
		4	superiortemporal	1.53	(54.7, -18.3, -3.4)	163386	0.0194
		5	precuneus	1.35	(17.0, -61.0, 28.7)	111719	0.0218
		6	insula	1.29	(39.0, 0.4, -20.4)	15531	0.0203
		7	middletemporal	1.22	(46.6, -61.0, 3.9)	25485	0.0257
		8	insula	1.21	(36.6, -9.0, -1.9)	102862	0.0249
Surface Area	Left	1	precuneus	1.82	(-16.5, -58.1, 18.8)	69338	0.0029
		2	superiortemporal	1.23	(-52.9, -19.8, 1.4)	155186	0.0058
		3	isthmuscingulate	1.11	(-11.6, -40.8, 32.9)	88276	0.0043
		4	superiorparietal	1.09	(-21.7, -84.5, 21.4)	98692	0.0197
	Right	1	precuneus	2.05	(15.5, -54.8, 17.7)	1675	0.0028
		2	superiorparietal	1.67	(30.2, -62.7, 28.0)	2334	0.0028
		3	lingual	1.64	(29.4, -52.4, -5.4)	822	0.0087
		4	posteriorcingulate	1.45	(14.8, -23.7, 37.1)	757	0.0028
		5	superiortemporal	1.13	(43.5, -30.2, 7.8)	28139	0.0028

Vertex-wise statistical analyses of significant group differences in left and right cortical metrics using the linear mixed effect model: $Cortical\,Metric \sim 1 + Group + Session + Group \times Session + (1 \mid Participant)$. Clusters were obtained through the FreeSurfer *mri_surfcluster* function using the False Discovery Rate (FDR) corrected p-value maps from one-tailed *Wald* F-tests. We used an α level of 5% and a minimum surface area of 1 cm^2. *Region* corresponds to the labels of the *Desikan-Killiany* atlas [52] , *size* corresponds to the surface area of the significant clusters while *MNI coordinates*, *vertex p min*, and *FDR-corrected p min* correspond to the peak with minimum p-value.

Table SM 6 Group comparison statistics of the global differences in cortical metrics at Pregnancy session ("Prg"), Postpartum session ("Post"), and "Prg-to-Post" in mothers and nulliparous women ("Controls") accounting for participant' age, intracranial volume, and mean *Euler* number.

	**Group differences**		
**Metric/Statistic**	**Prg**	**Post**	**Prg-to-Post**
**Cortical Volume [cm^3]**			
F-statistic	28.15	16.04	12.47
Degrees of freedom	1, 149.68	1, 149.61	1, 141.03
Uncorrected p-value	3.97e-07*	9.75e-05*	0.0006*
Signed effect size	−0.1583	−0.0968	0.0812
**Cortical Thickness [mm]**			
F-statistic	19.90	10.27	12.33
Degrees of freedom	1, 149.13	1, 149.06	1, 141.03
Uncorrected p-value	1.59e-05*	0.0017*	0.0006*
Signed effect size	−0.1178	−0.0644	0.0804
**Surface Area [cm^2]**			
F-statistic	1.13	0.41	6.98
Degrees of freedom	1, 141.77	1, 141.76	1, 141.00
Uncorrected p-value	0.2894	0.5225	0.0092*
Signed effect size	−0.0079	−0.0029	0.0472

The adjusted linear mixed effect model corresponds to $CorticalMetric \sim 1 + Group + Session + Age + Intracranial\ Volume + Euler\ Number + Group \times Session + (1 \mid Participant)$. Degrees of freedom were obtained using Satterthwaite's approximation. P-values below the threshold of 0.0001 are reported in exponential notation. Multiple comparisons were applied across metrics and contrasts. Asterisks indicate a False Discovery Rate-corrected p-value<0.05. Effect sizes were calculated as the signed partial eta squared (η_p^2) associated with the correspondent one-tailed *Wald* F-tests.

Table SM 7 Group comparison statistics of the global differences in cortical metrics at Pregnancy session ("Prg"), Postpartum session ("Post"), and "Prg-to-Post" in mothers and nulliparous women ("Controls") accounting for participant' age, intracranial volume, mean *Euler* number, sleep quality, and stress levels.

	**Group differences**		
**Metric/Statistic**	**Prg**	**Post**	**Prg-to-Post**
**Cortical Volume**			
F-statistic	28.36	15.74	12.36
Degrees of freedom	1, 154.43	1, 158.58	1, 141.40
Uncorrected p-value	3.50e-07*	0.0001*	0.0006*
Signed effect size	−0.1551	−0.0903	0.0804
**Cortical Thickness**			
F-statistic	20.23	10.13	12.47
Degrees of freedom	1, 153.55	1, 157.47	1, 141.28
Uncorrected p-value	1.35e-05*	0.0018*	0.0006*
Signed effect size	−0.1164	−0.0605	0.0811
**Surface Area**			
F-statistic	1.16	0.37	7.87
Degrees of freedom	1, 142.76	1, 143.62	1, 139.55
Uncorrected p-value	0.2828	0.5415	0.0057*
Signed effect size	−0.0081	−0.0026	0.0534

The adjusted linear mixed effect model corresponds to $CorticalMetric \sim 1 + Group + Session + Age + IntracranialVolume + EulerNumber + PSQI + PSS + Group \times Session + (1 \mid Participant)$ where PSQI corresponds to the global score of the Pittsburgh Sleep Questionnaire Index and PSS to the Perceived Stress Scale. Degrees of freedom were obtained using Satterthwaite's approximation. P-values below the threshold of 0.0001 are reported in exponential notation. Multiple comparisons were applied across metrics and contrasts. Asterisks indicate a False Discovery Rate-corrected p-value<0.05. Effect sizes were calculated as the signed partial eta squared (η_p^2) associated with the correspondent one-tailed *Wald* F-tests.

Table SM 8 Mothers' neuropsychological levels at late pregnancy and early postpartum sessions.

	Postpartum		Pregnancy		Session differences		
**Questionnaire**	**Mean**	**Standard Deviation**	**Mean**	**Standard Deviation**	**F-statistic**	**Uncorrected p-value**	**Signed effect size**
Perceived Stress (PSS)	20.48	9.72	15.88	8.08	42.84	2.00e-09*	0.282
Sleep Quality (PSQI)	10.36	3.22	8.18	3.84	33.61	6.66e-08*	0.236
Depression Symptoms (EDS)	6.05	4.99	4.20	3.75	19.70	2.17e-05*	0.153
Maternal Attachment (MAS)	80.08	7.08	71.76	5.92	178.42	0*	0.621
Pregnancy anxiety (PRAS)			62.96	12.10			
Maternal Stress (MSS)	26.55	6.95					
Birth Experience (BEQ)	3.27	0.92					

The adjusted linear mixed effect model corresponds to $Questionnaire\ Score \sim 1 + Session + (1 \mid Participant)$. Multiple comparisons were applied across questionnaires. Asterisks indicate a False Discovery Rate-corrected p-value<0.05. Effect sizes were calculated as the signed partial eta squared (η_p^2) associated with the correspondent one-tailed *Wald* F-tests. P-values below the threshold of 0.0001 are reported in exponential notation. Notice that the p-value associated with MAS is so small that the software data type precision rounds it down to 0. Abbreviations: PSS=Perceived Stress Scale, PSQI=Pittsbugh Sleep Quality Index, MAS=Maternal Attachment Scales, EDS=Edinburgh Depression Scales, PRAS=Pregnancy-related Anxiety Scale, MSS=Maternal Stress Scale, and BEQ=Birth Experience Questionnaire.

Table SM 9 Neuropsychological levels at Pregnancy session ("Prg"), Postpartum session ("Post"), and "Prg-to-Post" in mothers and nulliparous women ("Controls").

	Controls, N=34		Mothers, N=110		Group differences		
**Metric/Statistic**	**Prg**	**Post**	**Prg**	**Post**	**Prg**	**Post**	**Prg-to-Post**
**Sleep Quality (PSQI)**							
Mean	5.79	6.00	8.18	10.36			
Standard deviation	3.08	3.08	3.84	3.22			
F-statistic					12.55	41.91	7.67
Degrees of freedom					1, 237.53	1, 237.53	1, 142.00
Uncorrected p-value					0.0005*	5.43e-10*	0.0064*
Signed effect size					0.050	0.150	0.051
**Perceived Stress (PSS)**							
Mean	19.94	21.53	15.88	20.48			
Standard deviation	7.08	10.12	8.08	9.72			
F-statistic					5.43	0.36	4.71
Degrees of freedom					1, 193.57	1, 193.57	1, 142.00
Uncorrected p-value					0.0208*	0.5482	0.0317*
Signed effect size					−0.027	−0.002	0.032

The adjusted linear mixed effect model corresponds to $Questionnaire\ Score \sim 1 + Group + Session + Group \times Session + (1 \mid Participant)$. Degrees of freedom were obtained using Satterthwaite's approximation. Multiple comparisons were applied across metrics and contrasts. Asterisks indicate a False Discovery Rate-corrected p-value<0.05. Effect sizes were calculated as the signed partial eta squared (η_p^2) associated with the correspondent one-tailed *Wald* F-tests. P-values below the threshold of 0.0001 are reported in exponential notation. Abbreviations: Prg=Pregnancy session, Post=Postpartum session, PSS=Perceived Stress Scale, and PSQI=Pittsbugh Sleep Quality Index,

Table SM 10 Descriptives and group comparison statistics of the global differences in cortical metrics at Pregnancy session ("Prg"), Postpartum session ("Post"), and "Prg-to-Post" in "Labor" and "Pre-Labor" mothers.

	**Labor mothers, N=99**		**Pre-Labor mothers, N=11**		**Group differences**		
**Metric/Statistic**	**Prg**	**Post**	**Prg**	**Post**	**Prg**	**Post**	**Prg-to-Post**
**Cortical Volume [cm^3]**							
Mean	448.44	450.64	442.61	453.69			
Standard deviation	29.29	29.23	32.36	35.38			
F-statistic					0.38	0.11	24.20
Degrees of freedom					1, 109.99	1, 109.99	1, 108.00
Uncorrected p-value					0.5374	0.7464	3.13e-06*
Signed effect size					−0.0035	0.001	0.1831
**Cortical Thickness [mm]**							
Mean	2.41	2.42	2.39	2.43			
Standard deviation	0.07	0.06	0.08	0.07			
F-statistic					1.07	0.29	19.27
Degrees of freedom					1, 115.12	1, 115.12	1, 108.00
Uncorrected p-value					0.3021	0.5930	2.66e-05*
Signed effect size					−0.0092	0.0025	0.1514
**Surface Area [cm^2]**							
Mean	1656.61	1658.22	1648.78	1660.82			
Standard deviation	108.79	108.19	125.97	133.11			
F-statistic					0.05	0.01	13.40
Degrees of freedom					1, 108.36	1, 108.36	1, 108.00
Uncorrected p-value					0.8238	0.9410	0.0004*
Signed effect size					−0.0005	0.0001	0.1103

The adjusted linear mixed effect model corresponds to $Cortical Metric \sim 1 + Labor + Session + Labor \times Session + (1 \mid Participant)$. Degrees of freedom were obtained using Satterthwaite's approximation. Multiple comparisons were applied across metrics and contrasts. Asterisks indicate a False Discovery Rate-corrected p-value<0.05. Effect sizes were calculated as the signed partial eta squared (η_p^2) associated with the correspondent one-tailed *Wald* F-tests. P-values below the threshold of 0.0001 are reported in exponential notation.

Table SM 11 Group comparison statistics of the global differences in cortical metrics at Pregnancy session ("Prg"), Postpartum session ("Post"), and "Prg-to-Post" in "Labor" and "Pre-Labor" mothers accounting for participant' age, intracranial volume, mean *Euler* number, gestational weeks at childbirth, and the time between childbirth and the postpartum session.

	**Labor vs. Pre-labor differences in mothers**		
**Metric/Statistic**	**Prg**	**Post**	**Prg-to-Post**
**Cortical Volume**			
F-statistic	1.42	0.10	21.05
Degrees of freedom	1, 109.55	1, 109.47	1, 107.67
Uncorrected p-value	0.2360	0.7479	1.22e-05*
Signed effect size	−0.0128	0.0009	0.1635
**Cortical Thickness**			
F-statistic	1.83	0.00	18.53
Degrees of freedom	1, 109.17	1, 109.10	1, 107.63
Uncorrected p-value	0.1793	0.9833	3.70e-05*
Signed effect size	−0.0165	0	0.1469
**Surface Area**			
F-statistic	0.03	0.10	11.37
Degrees of freedom	1, 105.03	1, 105.01	1, 107.15
Uncorrected p-value	0.8721	0.7471	0.0010*
Signed effect size	−0.0002	0.001	0.0959

The adjusted linear mixed effect model corresponds to $CorticalMetric \sim 1 + Labor + Session + Age + IntracranialVolume + EulerNumber + PostpartumDays + GestationalWeeks + Labor \times Session + (1 \mid Participant)$. Degrees of freedom were obtained using Satterthwaite's approximation. Multiple comparisons were applied across metrics and contrasts. Asterisks indicate a False Discovery Rate-corrected p-value<0.05. Effect sizes were calculated as the signed partial eta squared (η_p^2) associated with the correspondent one-tailed *Wald* F-tests. P-values below the threshold of 0.0001 are reported in exponential notation.

Table SM 12 Descriptives and group comparison statistics of the global differences in cortical metrics at Pregnancy-to-Postpartum ("Prg-to-Post") between mothers based on the delivery method.

	Vaginal		Emergency C-section		Scheduled C-section		Prg-to-Post group comparisons		
**Metric/statistic**	**Prg**	**Post**	**Prg**	**Post**	**Prg**	**Post**	**Vaginal vs. Emergency C-section**	**Vaginal vs. Scheduled C-section**	**Emergency vs. Scheduled C-sections**
**Cortical Volume** [cm^3]									
Mean	446.50	448.80	462.53	463.97	442.61	453.69			
Standard deviation	27.97	27.76	35.77	36.94	32.36	35.38			
F-statistic							0.24	23.15	16.41
Degrees of freedom							1, 107.00	1, 107.00	1, 107.00
Uncorrected p-value							0.6244	4.94e-06*	9.69e-05*
Signed effect size							−0.0022	0.1779	−0.133
**Cortical Thickness** [mm]									
Mean	2.41	2.42	2.40	2.41	2.39	2.43			
Standard deviation	0.07	0.06	0.07	0.08	0.08	0.07			
F-statistic							1.51	17.86	17.20
Degrees of freedom							1, 107.00	1, 107.00	1, 107.00
Uncorrected p-value							0.2214	5.02e-05*	6.76e-05*
6.76e-05 Signed effect size							−0.0139	0.143	−0.1385
**Surface Area** [cm^2]									
Mean	1647.87	1649.35	1719.98	1722.51	1648.78	1660.82			
Standard deviation	102.88	102.10	133.09	132.89	125.97	133.11			
F-statistic							0.14	13.43	6.41
Degrees of freedom							1, 107.00	1, 107.00	1, 107.00
Uncorrected p-value							0.7073	0.0004*	0.0128*
Signed effect size							0.0013	0.1115	−0.0565

The adjusted linear mixed effect model corresponds to $Cortical\ Metric \sim 1 + BirthType + Session + BirthType \times Session + (1 \mid Participant)$. Degrees of freedom were obtained using Satterthwaite's approximation. Multiple comparisons were applied across metrics and contrasts. Asterisks indicate a False Discovery Rate-corrected p-value<0.05. Effect sizes were calculated as the signed partial eta squared (η_p^2) associated with the correspondent one-tailed *Wald* F-tests. P-values below the threshold of 0.0001 are reported in exponential notation.

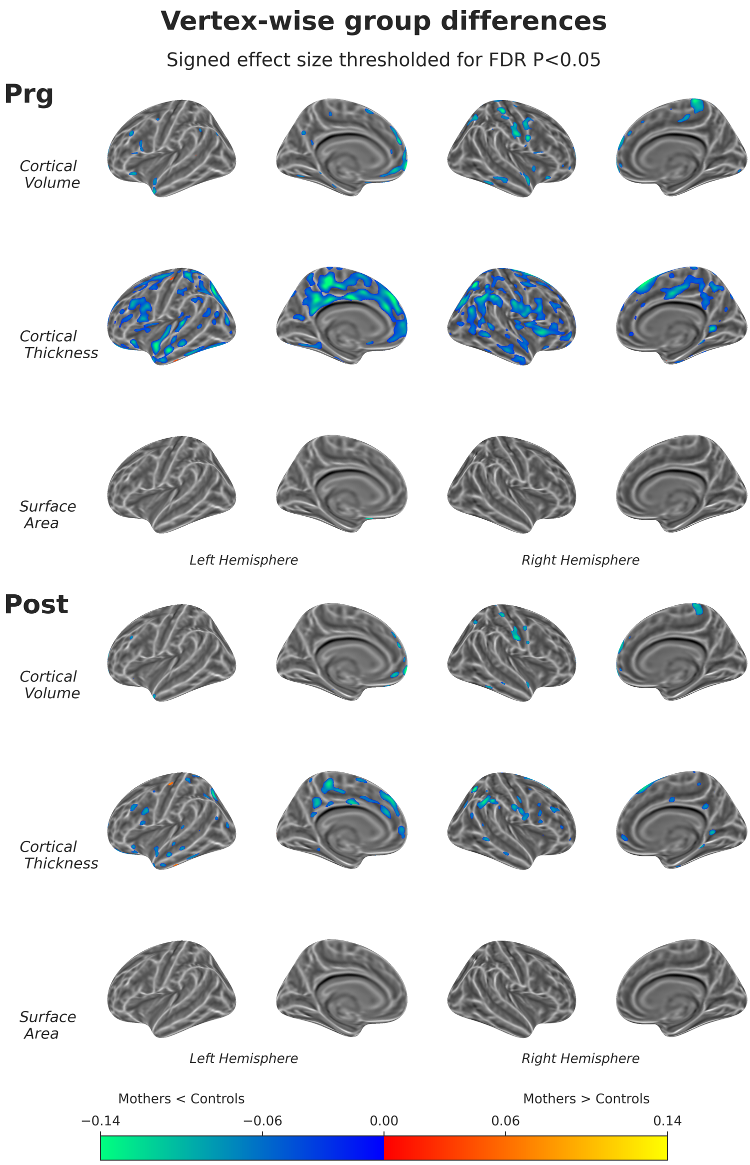

Fig. SM 1 Vertex-wise signed effect size maps of the significant cortical group differences (p<0.05, False Discovery Rate corrected) at "Prg" and "Post" sessions. Group fixed effects at each session were studied through the adjusted linear mixed model $Cortical\ Metric \sim 1{+}Group{+}Session{+}Group{\times}Session{+}(1 \mid Participant)$. Effect sizes of the group differences at "Prg" and "Post" sessions were calculated as the *partial eta squared* (η_p^2) associated to the correspondent one-tailed *Wald* F-tests. Cold and warm colors indicate lower and higher cortical values, respectively, in mothers compared to controls. Maps were projected to the inflated *fsaverage* template provided by the FreeSurfer software. Abbreviations: FDR=False Discovery Rate, Post=postpartum session, Prg=pregnancy session.

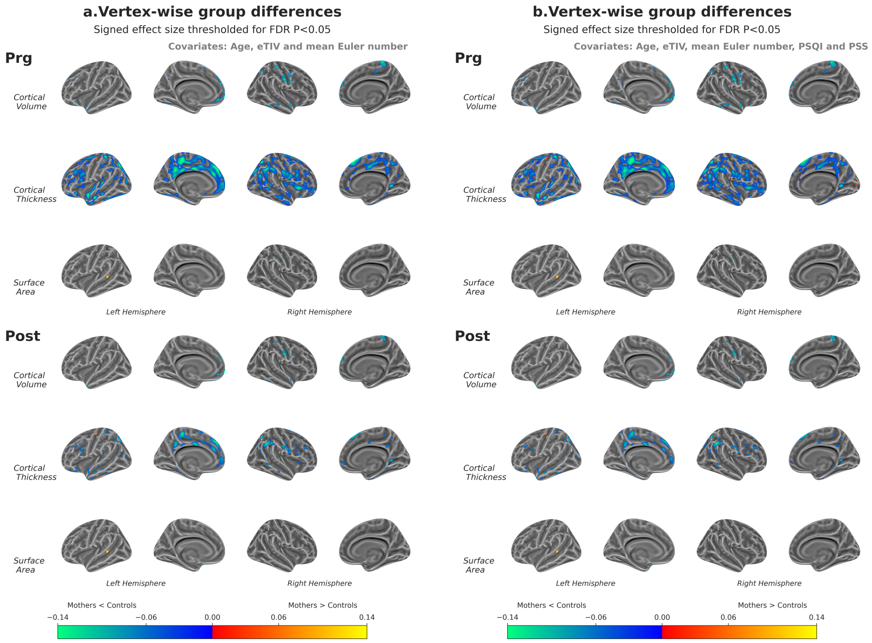

Fig. SM 2 Vertex-wise signed effect size maps of the significant cortical group differences ($p<0.05$, False Discovery Rate corrected) at "Prg" and "Post" sessions including potential confounding variables. Group fixed effects at each session were studied through the adjusted linear mixed effect model $Cortical : Metric \sim 1 + Group + Session + Group \times Session + Confoundings + (1 \mid Participant)$. Confoundings for models in (a) correspond to age, estimated total intracranial volume, and mean *Euler* number while for models in (b) correspond to age, estimated total intracranial volume, mean *Euler* number, Pittsburgh Sleep Quality Index, and Perceived Stress Scale. Effect sizes were calculated as the signed *partial eta squared* (η_p^2) associated to the correspondent one-tailed *Wald* F-tests. Cold and warm colors indicate lower and higher cortical values, respectively, in mothers compared to controls. Maps were projected to the inflated *fsaverage* template provided by the FreeSurfer software. Abbreviations: eTIV= estimated Total Intracranial Volume, FDR=False Discovery Rate, Post=postpartum session, Prg=pregnancy session, PSQI= Pittsburgh Sleep Quality Index, and PSS= Perceived Stress Scale.

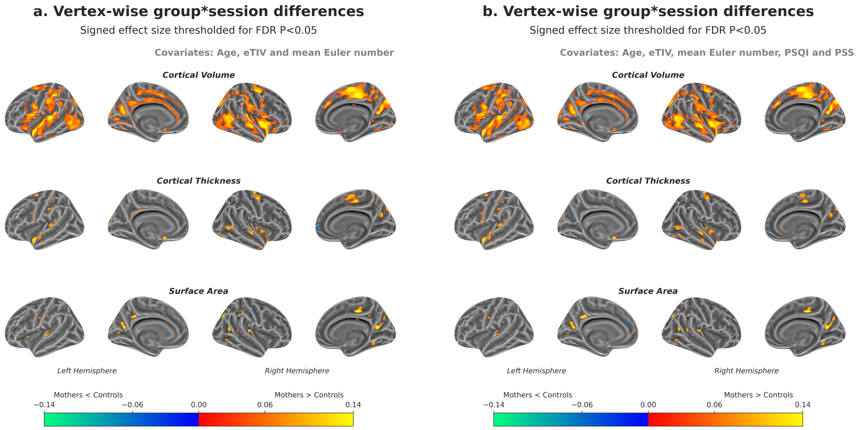

Fig. SM 3 Vertex-wise signed effect size maps for significant longitudinal changes in cortical metrics (p<0.05 False Discovery Rate corrected) including potential confounding variables. Longitudinal changes were derived from the group*session interaction fixed effect term of the adjusted linear mixed effect model $Cortical : Metric \sim 1 + Group + Session + Group \times Session + Confoundings + (1 \mid Participant)$. Confoundings for models in (a) correspond to age, estimated total intracranial volume, and mean *Euler* number while for models in (b) correspond to age, estimated total intracranial volume, mean *Euler* number, Pittsburgh Sleep Quality Index, and Perceived Stress Scale. Effect sizes were calculated as the signed *partial eta squared* (η_p^2) associated with the correspondent one-tailed *Wald* F-tests. Cold and warm colors indicate lower and higher cortical values, respectively, in mothers compared to controls. Maps were projected to the inflated *fsaverage* template provided by the FreeSurfer software. Abbreviations: eTIV= estimated Total Intracranial Volume, FDR=False Discovery Rate, Post=postpartum session, Prg=pregnancy session, PSQI= Pittsburgh Sleep Quality Index, and PSS= Perceived Stress Scale.

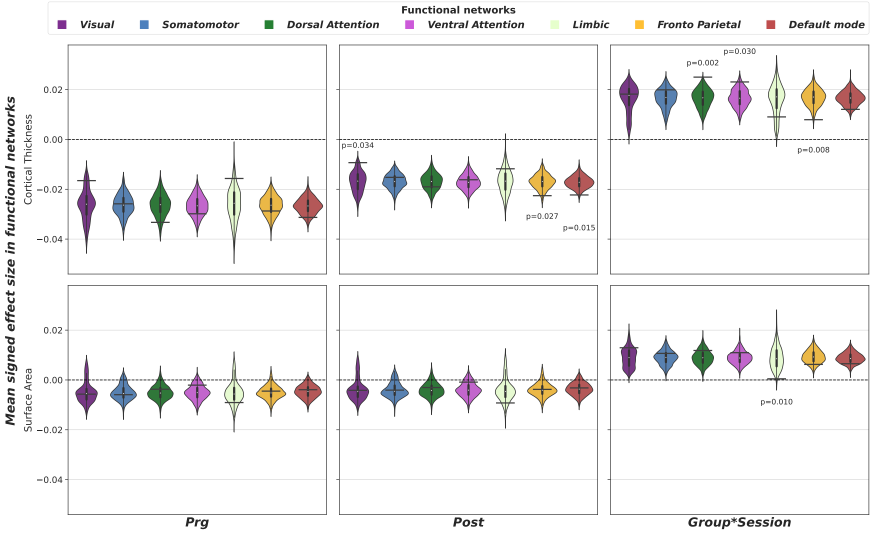

Fig. SM 4 Spin testing for the mean signed effect sizes of the vertex-wise group differences (mothers [N=110] compared to nulliparous women ["Controls", N=34]) in cortical thickness and surface area within the seven large-scale functional brain networks [19]. Black horizontal bars represent the observed values, whereas the violin plots reflect the null distributions obtained using 1000 spin-permutations of the maps. The exact one-tailed p-values are reported when $p<0.05$. No multiple comparisons corrections were applied. The white dot on the center of the boxplot represents the median, the box encloses the lower and upper quartiles, and the whiskers extend to the minimum and maximum values within a range of 1.5 times the interquartile range. Abbreviations: Post=postpartum session, Prg=pregnancy session, Prg-to-Post=from pregnancy to postpartum sessions.

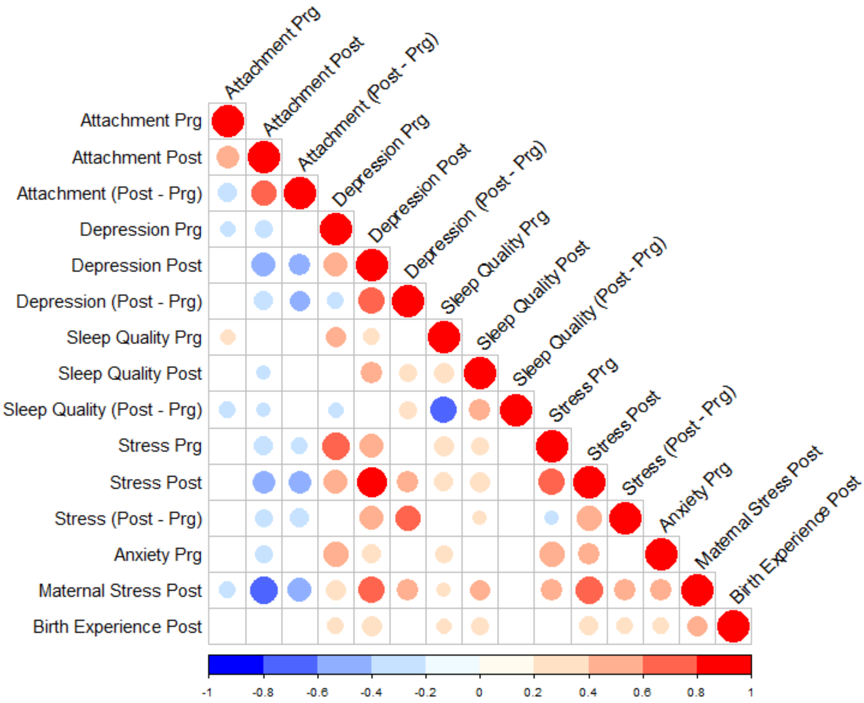

Fig. SM 5 Complete correlation matrix of the neuropsychological variables in the mothers (N=110), including the scales at the late pregnancy ("Prg") and early postpartum ("Post") sessions. Circles represent the two-tailed Pearson coefficients of those correlations surviving a p<0.05 False-Discovery Rate correction. Blue and red circles indicate negative and positive correlations, respectively. From top to bottom, the neuropsychological variables correspond to the following questionnaires: Maternal Attachment Scale, Edinburgh Depression Scale, Pittsburgh Sleep Quality Index, Perceived Stress Scale, Pregnancy Anxiety Scale, Maternal Stress Scale, and Birth Experience Questionnaire.

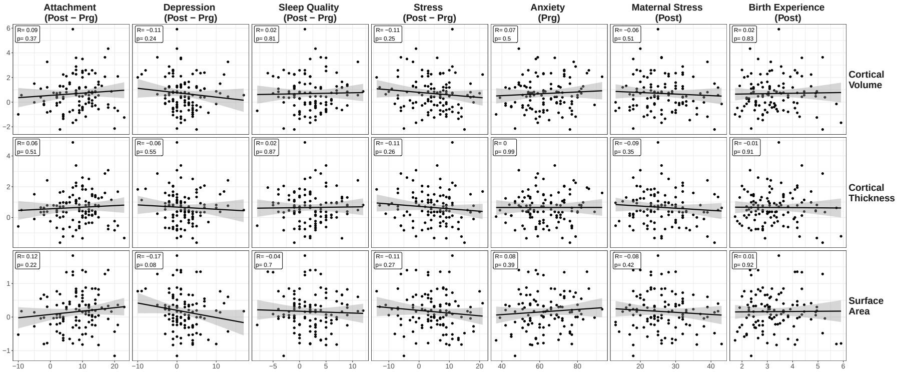

Fig. SM 6 Correlation matrix between the mothers' global percentages of change in cortical metrics and the neuropsychological variables. From left to right, the neuropsychological variables correspond to the following questionnaires: Maternal Attachment Scale, Edinburgh Depression Scale, Pittsburgh Sleep Quality Index, Perceived Stress Scale, Pregnancy Anxiety Scale, Maternal Stress Scale, and Birth Experience Questionnaire. The black line and the shaded area represent the least squares regression line and the 95% confidence interval. Abbreviations: p=uncorrected p-value, Post=Postpartum session, Prg=Pregnancy session, R=two-tailed *Pearson* correlation coefficient.

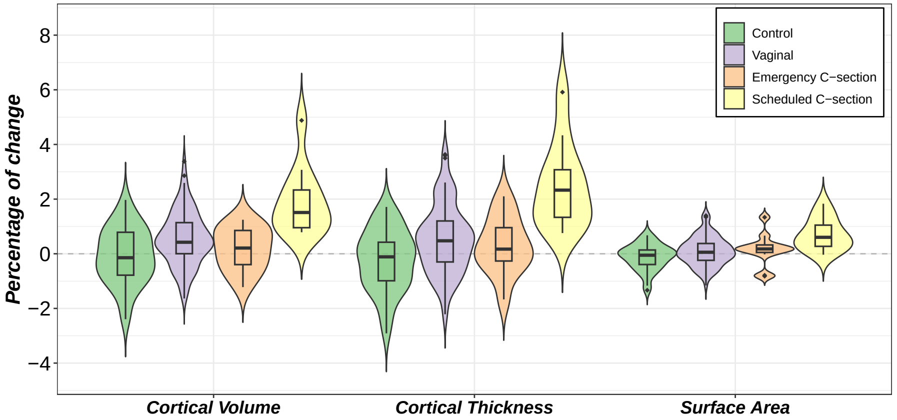

Fig. SM 7 Global percentages of change in cortical volume, thickness, and surface area in mothers who underwent a vaginal (N=87), emergency cesarean section (C-section, N=12), or scheduled C-section (N=11). Nulliparous women ("Control", N=34) are displayed as a reference. The center line of the boxplot represents the median, the box encloses the lower and upper quartiles, and the whiskers extend to the minimum and maximum values within a range of 1.5 times the interquartile range.

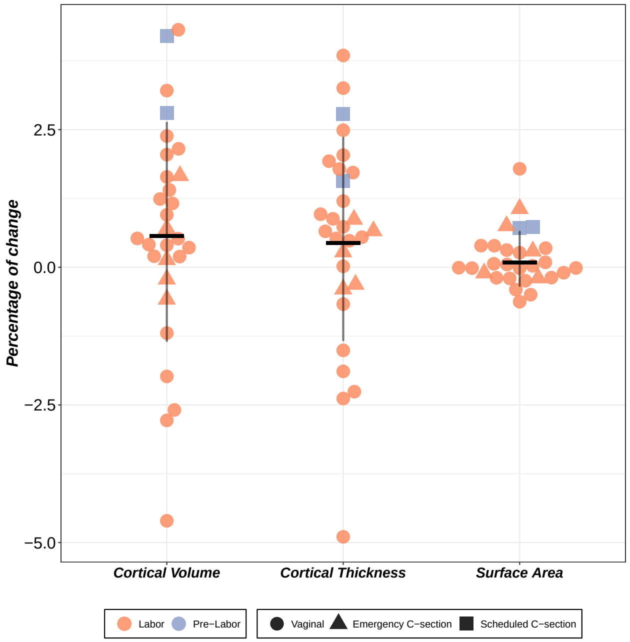

Fig. SM 8 Global percentages of change in cortical volume, thickness, and surface area in mothers (N=29) of the replication sample. Orange circles represent mothers who underwent a vaginal delivery (N=22), orange triangles show those who experienced emergency cesarean section (C-section, N=5), and purple squares represent those mothers with scheduled C-sections (N=2). Black horizontal lines represent the mean percentages of change values for the whole mothers' sample and vertical lines their standard deviation.

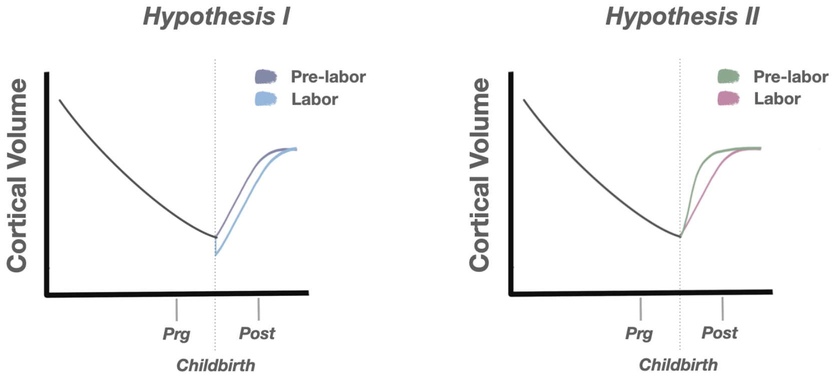

Fig. SM 9 Hypothesized trajectories of cortical volume as a function of labor. Hypothesis I: Mothers experiencing at least the first stage of labor ("Labor") may undergo further cortical reductions, reaching a lower cortical volume before reversing the trajectory. Hypothesis II: Mothers who did not initiate labor ("Pre-labor") may have a faster neural recovery.

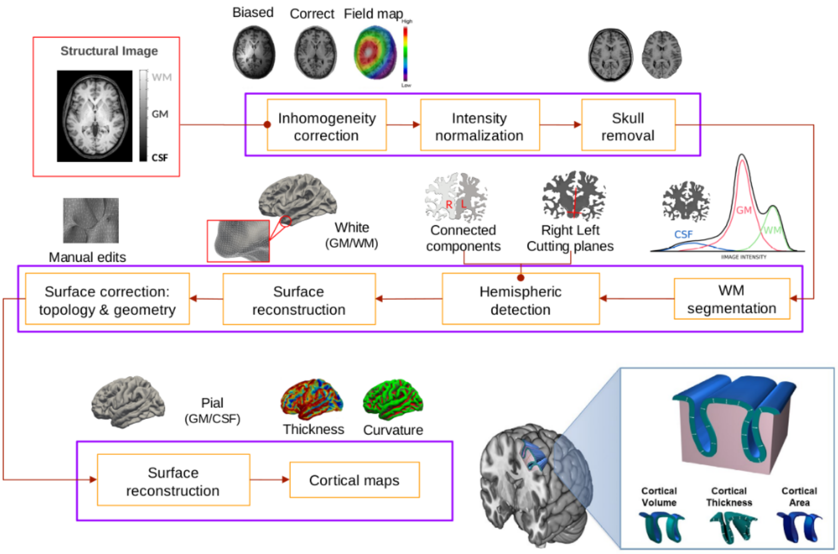

Fig. SM 10 Schematic representation of the FreeSurfer's cross-sectional pipeline.

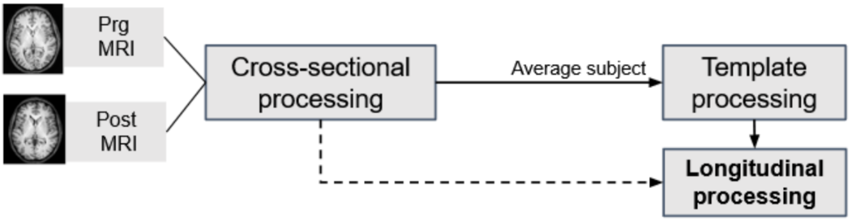

Fig. SM 11 Schematic representation of the FreeSurfer's longitudinal pipeline.

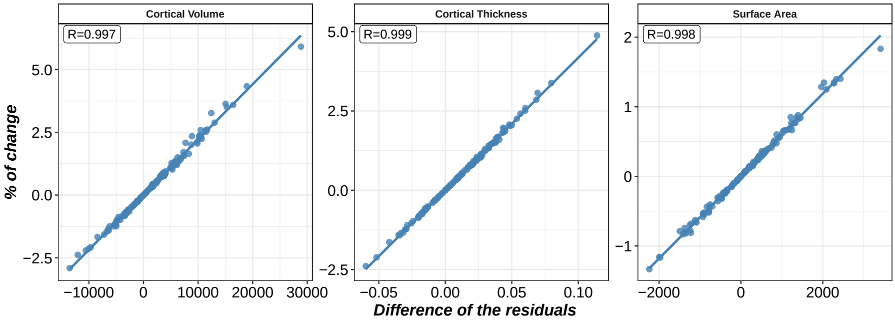

Fig. SM 12 *Pearson* (R) correlation between the percentage of change in cortical metrics and the difference of the residuals obtained from the reduced linear mixed effect models.
