# Supplementary material for: Women’s neuroplasticity during gestation, childbirth and postpartum
Source: Nat Neurosci. 2024 Jan 5;27(2):319–27. doi: 10.1038/s41593-023-01513-2 (PMC10849958; doi:10.1038/s41593-023-01513-2)
Supplement: Supplementary file 2 — Reporting Summary [file 41593_2023_1513_MOESM2_ESM.pdf]

nature portfolio

Corresponding author(s): Susana Carmona

Last updated by author(s): Oct 23, 2023

Reporting Summary

Nature Portfolio wishes to improve the reproducibility of the work that we publish. This form provides structure for consistency and transparency in reporting. For further information on Nature Portfolio policies, see our Editorial Policies and the Editorial Policy Checklist.

Statistics

For all statistical analyses, confirm that the following items are present in the figure legend, table legend, main text, or Methods section.

n/a	Confirmed	
☐	☒	The exact sample size (*n*) for each experimental group/condition, given as a discrete number and unit of measurement
☐	☒	A statement on whether measurements were taken from distinct samples or whether the same sample was measured repeatedly
☐	☒	The statistical test(s) used AND whether they are one- or two-sided<br>*Only common tests should be described solely by name; describe more complex techniques in the Methods section.*
☐	☒	A description of all covariates tested
☐	☒	A description of any assumptions or corrections, such as tests of normality and adjustment for multiple comparisons
☐	☐	A full description of the statistical parameters including central tendency (e.g. means) or other basic estimates (e.g. regression coefficient) AND variation (e.g. standard deviation) or associated estimates of uncertainty (e.g. confidence intervals)
☐	☒	For null hypothesis testing, the test statistic (e.g. *F*, *t*, *r*) with confidence intervals, effect sizes, degrees of freedom and *P* value noted<br>*Give P values as exact values whenever suitable.*
☒	☐	For Bayesian analysis, information on the choice of priors and Markov chain Monte Carlo settings
☒	☐	For hierarchical and complex designs, identification of the appropriate level for tests and full reporting of outcomes
☐	☒	Estimates of effect sizes (e.g. Cohen's *d*, Pearson's *r*), indicating how they were calculated

Our web collection on statistics for biologists contains articles on many of the points above.

Software and code

Policy information about availability of computer code

Data collection	Qualtrics XM, Siemens MAGNETOM Vida 3 Tesla (main dataset), and Philips Ingenia CX 3 Tesla (replication dataset).
Data analysis	MRI preprocessing was performed with Freesurfer version 7.1.1 (main dataset) and 7.2 (replication dataset).<br><br>Statistical analyses were performed in Rstudio (version 2022.02.3+294), under R version 4.2.1, with the following libraries: fslmer (version 0.0.0.9002) for LME models, including global, vertex-wise, and neuropsychological data and stats (version 4.2.1) for correlations and FDR correction. Vertex-wise FDR-corrected maps were computed using FSL fdr function (version 6.0.5). The clusters' location of the vertex-wise results were obtained using the FreeSurfer's mri surfcluster function (version 7.2.0). Spin tests were performed using the spin-test toolbox (https://github.com/spin-test/spin-test) and the following Python (version 3.9.7) packages: nibabel (version 3.2.1), pandas (version 1.5.3), numpy (version 1.21.5), and scipy (version 1.10.1).<br><br>Figures were plotted using ggplot2 (version 3.3.6), GGally (version 2.1.2), ggpubr (version 0.4.0), and corrplot (version 0.9.2) R libraries. Vertex-wise analyses were plotted using the nilearn (version 0.9.1), nibabel (version 3.2.1), and matplotlib (version 3.6.2) Python packages.<br><br>All the data and code necessary to replicate and extend our findings are available in the following GitHub repository: https://github. com/ neuromaternal/peripartum neuroplasticity.

For manuscripts utilizing custom algorithms or software that are central to the research but not yet described in published literature, software must be made available to editors and reviewers. We strongly encourage code deposition in a community repository (e.g. GitHub). See the Nature Portfolio guidelines for submitting code & software for further information.

Data

Policy information about availability of data

All manuscripts must include a data availability statement. This statement should provide the following information, where applicable:

- Accession codes, unique identifiers, or web links for publicly available datasets
- A description of any restrictions on data availability
- For clinical datasets or third party data, please ensure that the statement adheres to our policy

The datasets including the global cortical metrics, demographic information, obstetric data, and neuropsychological information generated and analyzed in the current study are available in the GitHub repository (https://github.com/neuromaternal/peripartum_neuroplasticity). Effect sizes and significance vertex-wise maps reported in the manuscript are also available there.
Thus, all the data and code necessary to replicate and extend our findings are available in the repository. The transfer of the raw and processed MRI images of the study participants requires additional data treatment agreement including the purpose of the use, and thus, are only available upon reasonable request to the corresponding author.

Research involving human participants, their data, or biological material

Policy information about studies with human participants or human data. See also policy information about sex, gender (identity/presentation), and sexual orientation and race, ethnicity and racism.

Reporting on sex and gender	The subjects of this study were pregnant and non-pregnant females that self-identified as women. Therefore, sex- and gender-based analyses were not applicable.<br>We disclosed the sex and gender terminology used across the manuscript with the following statement "In this manuscript, we use the term "women" to refer to females whose sex matches their gender and "mothers" to refer to females that were pregnant and gave birth to their children, in keeping with current practices in the field of parental neuroscience. This terminology will need to evolve to be more inclusive as the field expands to also include gestational people whose sex and gender do not match."
Reporting on race, ethnicity, or other socially relevant groupings	No information with regard to race or ethnicity was collected.
Population characteristics	Elegible subjects were adult females in their reproductive years, either females undergoing their first pregnancy (main dataset: mean ± sd=33.12±3.98 years; replication dataset: mean±sd age=32.74±3.76 years) or non-pregnant nulliparous females (main dataset: mean±sd=33.32±4.56 years; replication dataset: mean±sd=34.32±0.84 weeks).<br>Among the pregnant women of the main dataset, 87 underwent vaginal delivery (mean±sd age=33.11±3.99 years), 12 emergency c-section (mean±sd age=34.13±4.25 years), and 11 scheduled c-section (mean±sd age=32.11±3.65 years).<br><br>Exclusion criteria were as follows: an estimated intelligence quotient below 80 (estimated by Wechsler Adult Intelligence Scale (WAIS-IV) Digit Span subtest scores), previous pregnancies beyond the first trimester, being a foster parent, gestating twins, past or current neurological disorders, and past or current psychiatric conditions as assessed by the MINI International Neuropsychiatric Interview.
Recruitment	Participant recruitment was performed through word-of-mouth, health perinatal professionals, and the research group's social media channels. Candidates that expressed interest in participating contacted us via email or personally. We are not aware of specific self-selection biases that a priori could affect the results. We are confident that the study's participants constitute a reliable representation of the population of first-time mothers and nulliparous women in Madrid and Barcelona (Spain). However, it's important to note that this population may be somewhat limited in its representation of the diverse races, ethnicities and cultural backgrounds found in the global population.
Ethics oversight	The study was conducted in accordance with the Declaration of Helsinki and Good Clinical Practice guidelines. The protocol was approved by the Ethics Committee of each center (Instituto de Investigacion Sanitaria del Hospital Gregorio Marañon, Madrid, Spain (main dataset) and Universitat Autonoma de Barcelona, Barcelona, Spain (replication dataset)). All participants signed a consent form before participating in the study.

Note that full information on the approval of the study protocol must also be provided in the manuscript.

Field-specific reporting

Please select the one below that is the best fit for your research. If you are not sure, read the appropriate sections before making your selection.

☐ Life sciences ☒ Behavioural & social sciences ☐ Ecological, evolutionary & environmental sciences

For a reference copy of the document with all sections, see nature.com/documents/nr-reporting-summary-flat.pdf

Behavioural & social sciences study design

All studies must disclose on these points even when the disclosure is negative.

Study description	We used a quantitative longitudinal case-control study assessing first-time mothers (N=110) at late pregnancy and early postpartum and a control group of nulliparous women (N=34). The main results were tested in an independent sample formed of 29 mothers and 24 controls.
Research sample	Main dataset: 111 first-time pregnant women were assessed at the end of the third trimester of pregnancy (mean ± sd = 36.23 ± 0.96 weeks). Of these women, 110 returned to a second session at the first month postpartum (mean ± sd = 22 ± 8 days), when they were allocated as having either initiated labor or undergone a scheduled cesarean section, depending on their birth type. As a control group, 36 age-matched non-pregnant nulliparous women took part in the initial session. Of these women, 34 returned to the second session at a time interval equivalent to that of the mothers' group (mean ± sd = 44 ± 10 days).<br>Replication dataset: 35 first-time pregnant women were assessed at the end of the third trimester of pregnancy (mean ± sd = 34.32 ± 0.84 weeks). Of these women, 29 returned to a second session at the first month postpartum (mean ± sd = 33.1 ± 5.8 days). As a control group, 24 age-matched non-pregnant nulliparous women took part in the initial session. All of them returned to the second session at a time interval equivalent to that of the mothers' group (mean ± sd = 73.53 ± 11.73 days).<br>Only participants who had the two sessions were included in the analyses.<br>The study sample was in their thirties (age range 24-46 years) and is representative of the Spanish pregnant population.
Sampling strategy	Sampling of participants was done using different strategies, including snowball sampling, voluntary response sampling and stratified sampling.<br>Final sample size of the main dataset was based on previous longitudinal studies of the field aiming for the largest possible sample size while working within funding-related constraints. The sample size of the pregnant women was determined based on the percentage of mothers estimated to undergo a cesarean section delivery (19 % of mothers, Candel et al 2020).<br>Vila-Candel, R., Martín, A., Escuriet, R., Castro-Sánchez, E., & Soriano-Vidal, F. J. (2020). Analysis of caesarean section rates using the robson classification system at a university hospital in Spain. International Journal of Environmental Research and Public Health, 17(5), 1575.
Data collection	MRI data: Siemens Magnetom Vida 3T MRI scanner (main dataset) and Philips Ingenia CX 3T MRI scanner (replication dataset)<br>Demographic, obstetric and neuropsychological questionnaires: auto-administered via Qualtrics.<br>WAIS IV digits: in-visit administration by a clinical psychologist.<br>MINI international neuropsychiatric interview: administered by phone by a clinical psychologist.<br><br>The study visits included the participant, three researchers, a clinical psychologist, and an MRI technician. Due to the objectives and design of the study, data collection and analysis were not performed blind to the conditions of the experiments.
Timing	Data collection started 08-12-2020 and ended 01-25-2023.
Data exclusions	We performed a visual check on site and repeated the acquisition when artifacts were present. All collected anatomical scans could be used for the analysis.
Non-participation	Three participants from the main dataset (one mother and two nulliparous women) and six participants from the replication dataset (all mothers) did not return for the second session due to loss of interest. These participants were not included in the analysis.
Randomization	Randomization is not applicable to this study, since group allocation was decided upon the status of the participants (pregnant and non-pregnant).

Reporting for specific materials, systems and methods

We require information from authors about some types of materials, experimental systems and methods used in many studies. Here, indicate whether each material, system or method listed is relevant to your study. If you are not sure if a list item applies to your research, read the appropriate section before selecting a response.

Materials & experimental systems

n/a	Involved in the study	
☒	☐	Antibodies
☒	☐	Eukaryotic cell lines
☒	☐	Palaeontology and archaeology
☒	☐	Animals and other organisms
☒	☐	Clinical data
☒	☐	Dual use research of concern
☒	☐	Plants

Methods

n/a	Involved in the study	
☒	☐	ChIP-seq
☒	☐	Flow cytometry
☐	☒	MRI-based neuroimaging

Plants

Seed stocks | *Report on the source of all seed stocks or other plant material used. If applicable, state the seed stock centre and catalogue number. If plant specimens were collected from the field, describe the collection location, date and sampling procedures.*

Novel plant genotypes | *Describe the methods by which all novel plant genotypes were produced. This includes those generated by transgenic approaches, gene editing, chemical/radiation-based mutagenesis and hybridization. For transgenic lines, describe the transformation method, the number of independent lines analyzed and the generation upon which experiments were performed. For gene-edited lines, describe the editor used, the endogenous sequence targeted for editing, the targeting guide RNA sequence (if applicable) and how the editor was applied.*

Authentication | *Describe any authentication procedures for each seed stock used or novel genotype generated. Describe any experiments used to assess the effect of a mutation and, where applicable, how potential secondary effects (e.g. second site T-DNA insertions, mosiacism, off-target gene editing) were examined.*

Magnetic resonance imaging

Experimental design

Design type | High-resolution T1-weigthed anatomical Magnetic Resonance Imaging

Design specifications | Since this is a structural MRI sequence, no blocks, trials, or experimental units are applicable.

Behavioral performance measures | Since this is a structural MRI sequence, behavioral performances are not applicable.

Acquisition

Imaging type(s) | Structural

Field strength | 3T

Sequence & imaging parameters | Main Sample: We used a magnetization prepared rapid gradient-echo (MPRAGE) sequence in sagittal orientation with the following parameters: Voxel size=0.9375x0.9375x1 mm3; Field of View (FOV)=240x240x176 mm3; Echo Time (TE)=44 ms; Repetition Time (TR)=9.8/2300ms; Inversion Time (TI)=900ms; Flip Angle=8º; GRAPPA Acceleration Factor=2; Percent Sampling=80; Acquisition Time=265s.
Replication sample: We used a Turbo Field Echo (TFE) sequence in sagittal orientation and the following parameters: Voxel size=0.75x0.75x1mm3; FOV=240x240x180mm3; TE=46ms; TR=9.9/2300ms; Prepulse Delay=900ms; FA=8º; Acceleration factor=1.9; Percent Sampling=78%; Acquisition Time=259s.

Area of acquisition | Whole brain

Diffusion MRI ☐ Used ☒ Not used

Preprocessing

Preprocessing software | FreeSurfer (main dataset V 7.1.1, replication dataset V 7.2.0) longitudinal stream (https://surfer.nmr.mgh.harvard.edu/fswiki/LongitudinalProcessing), which comprises a cross-sectional processing of the images, a creation of a base within-subject template, and a longitudinal processing of the images using the template.

Normalization | Global analyses:
-Normalization to create the within-subject template (non-linear spherical surface registration)
Vertex-wise analyses:
-Normalization to create the within-subject template (non-linear spherical surface registration)
-Normalization to fsaverage (non-linear spherical surface registration)

Normalization template | Global analyses: Within-subject space (native space)
Vertex-wise analyses: fsaverage space to achieve a vertex-correspondance among subjects

Noise and artifact removal | Freesurfer's recon-all inhomogeneity correction
The normalization to fsaverage was smoothed with a 10mm full-width-at-half-maximum Gaussian kernel filter

Volume censoring | Since this is a structural MRI sequence, volume censoring is not applicable.

Statistical modeling & inference

Model type and settings | Vertex-wise analysis: Linear mixed effect (LME) models with random intercepts with covariates

Effect(s) tested | We computed group differences using LME Models

Specify type of analysis: ☒ Whole brain ☐ ROI-based ☐ Both

Statistic type for inference

(See Eklund et al. 2016)

Vertex-wise

Correction

False Discovery Rate correction (alpha<5 %, P<0.05)

Models & analysis

n/a	Involved in the study
☒	☐ Functional and/or effective connectivity
☒	☐ Graph analysis
☒	☐ Multivariate modeling or predictive analysis
